# Advancing systemic toxicity risk assessment: Evaluation of a NAM-based toolbox approach

**DOI:** 10.1093/toxsci/kfae159

**Published:** 2024-12-18

**Authors:** Sophie Cable, Maria Teresa Baltazar, Fazila Bunglawala, Paul L Carmichael, Leonardo Contreas, Matthew Philip Dent, Jade Houghton, Predrag Kukic, Sophie Malcomber, Beate Nicol, Katarzyna R Przybylak, Ans Punt, Georgia Reynolds, Joe Reynolds, Sharon Scott, Dawei Tang, Alistair M Middleton

**Affiliations:** Safety and Environmental Assurance Centre (SEAC), Unilever, Colworth Science Park, Sharnbrook MK44 1lQ, United Kingdom; Safety and Environmental Assurance Centre (SEAC), Unilever, Colworth Science Park, Sharnbrook MK44 1lQ, United Kingdom; Safety and Environmental Assurance Centre (SEAC), Unilever, Colworth Science Park, Sharnbrook MK44 1lQ, United Kingdom; Safety and Environmental Assurance Centre (SEAC), Unilever, Colworth Science Park, Sharnbrook MK44 1lQ, United Kingdom; Safety and Environmental Assurance Centre (SEAC), Unilever, Colworth Science Park, Sharnbrook MK44 1lQ, United Kingdom; Safety and Environmental Assurance Centre (SEAC), Unilever, Colworth Science Park, Sharnbrook MK44 1lQ, United Kingdom; Safety and Environmental Assurance Centre (SEAC), Unilever, Colworth Science Park, Sharnbrook MK44 1lQ, United Kingdom; Safety and Environmental Assurance Centre (SEAC), Unilever, Colworth Science Park, Sharnbrook MK44 1lQ, United Kingdom; Safety and Environmental Assurance Centre (SEAC), Unilever, Colworth Science Park, Sharnbrook MK44 1lQ, United Kingdom; Safety and Environmental Assurance Centre (SEAC), Unilever, Colworth Science Park, Sharnbrook MK44 1lQ, United Kingdom; Safety and Environmental Assurance Centre (SEAC), Unilever, Colworth Science Park, Sharnbrook MK44 1lQ, United Kingdom; Safety and Environmental Assurance Centre (SEAC), Unilever, Colworth Science Park, Sharnbrook MK44 1lQ, United Kingdom; Safety and Environmental Assurance Centre (SEAC), Unilever, Colworth Science Park, Sharnbrook MK44 1lQ, United Kingdom; Safety and Environmental Assurance Centre (SEAC), Unilever, Colworth Science Park, Sharnbrook MK44 1lQ, United Kingdom; Safety and Environmental Assurance Centre (SEAC), Unilever, Colworth Science Park, Sharnbrook MK44 1lQ, United Kingdom; Safety and Environmental Assurance Centre (SEAC), Unilever, Colworth Science Park, Sharnbrook MK44 1lQ, United Kingdom; Safety and Environmental Assurance Centre (SEAC), Unilever, Colworth Science Park, Sharnbrook MK44 1lQ, United Kingdom

**Keywords:** new approach methodologies, risk assessment, systemic toxicity

## Abstract

For many years, a method that allowed systemic toxicity safety assessments to be conducted without generating new animal test data, seemed out of reach. However, several different research groups and regulatory authorities are beginning to use a variety of in silico, in chemico, and in vitro techniques to inform safety decisions. To manage this transition to animal-free safety assessments responsibly, it is important to ensure that the level of protection offered by a safety assessment based on new approach methodologies (NAMs), is at least as high as that provided by a safety assessment based on traditional animal studies. To this end, we have developed an evaluation strategy to assess both the level of protection and the utility offered by a NAM-based systemic safety “toolbox.” The toolbox comprises physiologically based kinetic models to predict internal exposures, and bioactivity NAMs designed to give broad coverage across many different toxicity modes of action. The output of the toolbox is the calculation of a bioactivity:exposure ratio (analogous to a margin of internal exposure), which can be used to inform decision-making. In this work, we have expanded upon an initial pilot study of 10 chemicals with an additional 38 chemicals and 70 consumer exposure scenarios. We found that, for the majority of these (>90%), the NAM-based workflow is protective of human health, enabling us to make animal-free safety decisions for systemic toxicity and preventing unnecessary animal use. We have also identified critical areas for improvement to further increase our confidence in the robustness of the approach.

A major challenge in the evaluation of nonanimal safety assessments is establishing that they are truly protective of human health. Chemical risk assessment for systemic toxicity has traditionally relied on the use of repeated-dose in vivo tests to derive protective thresholds, mostly based on nonspecific bioactivity observed in animals (Browne et al. 2024). These no-observed(-adverse)-effect-levels (NO(A)ELs) or benchmark dose levels (BMDLs) from chronic and subchronic animal tests are then compared with the estimated exposure in humans. If there is a sufficiently large margin between them, which is determined by using intra- and interspecies uncertainty factors (or other appropriate uncertainty factors arising from consideration of matrices differences, the exposure estimation etc), then the human exposure can be considered low-risk.

The objective of using bioactivity data to derive thresholds that are protective of human health remains the goal of next-generation risk assessment (NGRA) (Dent et al. 2018), i.e. chemical risk assessment that does not involve the generation of new animal data. Where there are data gaps in existing information, or for de novo risk assessments, NGRA assesses bioactivity by estimating points of departure (PoDs) from a range of different in vitro studies and compares them with internal exposure concentrations (e.g. plasma *C*_max_) to calculate a bioactivity:exposure ratio (BER), analogous to the calculation of a margin of safety/exposure (MoS/E).

The overall approach to such a tiered, nonanimal, exposure-led assessment (Fig. 1) has been described in the literature (Berggren et al. 2017; Thomas et al. 2019); adapted and incorporated into published NGRA case studies (Baltazar et al. 2020; OECD 2021; Ebmeyer et al. 2024; Wood et al. 2024). These case studies demonstrated that NGRA is a flexible approach to integrating multiple lines of evidence to reach a safety decision. However, in order to build confidence in new approach methodologies (NAMs) for systemic toxicity, there is a need to evaluate their robustness and performance in a systematic manner for a wide range of chemicals if they are to be considered for regulatory applications (Dent et al. 2021; Westmoreland et al. 2022). Recent validation frameworks emphasize the need for defining the context of use as a first step in establishing a validation strategy (Van der Zalm et al. 2022; ICCVAM 2024). In our previous work (Middleton et al. 2022), we outlined an approach to evaluating a NAM-based systemic-safety toolbox and workflow comprising bioactivity and exposure modules, which could form a basis for estimating BERs at Tier 1 of an NGRA framework (Fig. 1). Based on the proposed validation frameworks, the “context of use” for this toolbox is defined as an early-tier approach for conducting systemic toxicity risk assessments for consumer goods which aims at being protective of human health rather than predicting specific hazards, similar to the traditional approach utilising in vivo data (Browne et al. 2024). The systemic-safety toolbox is not intended to be used to predict genotoxicity, carcinogenicity or developmental, and reproductive toxicity. We published the pilot phase of a 2-step process suggested as a method for evaluating and integrating data from multiple NAMs. The aim of this 2-step process (a pilot study and present evaluation) was to remove potential bias that could occur due to postevent rationalization of the BERs determined and increase confidence in the results obtained. The systemic-safety toolbox comprises 3 bioactivity platforms (high-throughput transcriptomics [HTTr], in vitro pharmacological profiling [IPP], and a cellular stress panel [CSP]), from which a minimum PoD is derived. For a given exposure scenario, physiologically based kinetic (PBK) models are used to estimate the corresponding internal exposure, which is quantified using the maximum plasma concentration (*C*_max_). The minimum PoD and the *C*_max_ are then used to calculate a BER. The performance of the toolbox can therefore be determined by investigating its capability to identify high-risk scenarios as high-risk (i.e. make protective decisions) for which the outcome of an in vivo*-*based risk assessment is known and well-documented. As a proof of concept, the initial pilot study was conducted by selecting 10 well-known chemicals for which we could define exposure scenarios that are either considered high-risk from a consumer goods perspective (i.e. drugs at therapeutic doses that are systemically active) or low-risk from a consumer goods perspective (i.e. existing food or cosmetic ingredients within their accepted use levels). As expected, for these cases, the magnitude of the BER correlated well with risk and the high-risk chemical-exposure scenarios were associated with smaller BERs than the low-risk chemical-exposure scenarios. The limited training set of chemical-exposure scenarios (*n* = 24) indicated that, depending on whether the exposure estimates were provided by a PBK model parameterized with in silico only, in vitro, or human in vivo data, a BER of 110, 11, or 2.5 or greater, respectively, was needed to confidently conclude a low-risk outcome. The reason for these different BER thresholds is the reduced uncertainty associated with more refined exposure models. Using a threshold of 11 (from a PBK model parameterized with in vitro data) decisions made using the systemic-safety toolbox were protective for 100% of high-risk chemical-exposure scenarios (protectiveness: 5 out of 5), while correctly identifying 69% of the low-risk scenarios as low-risk (utility: 9 out of 13).

**Fig. 1.**
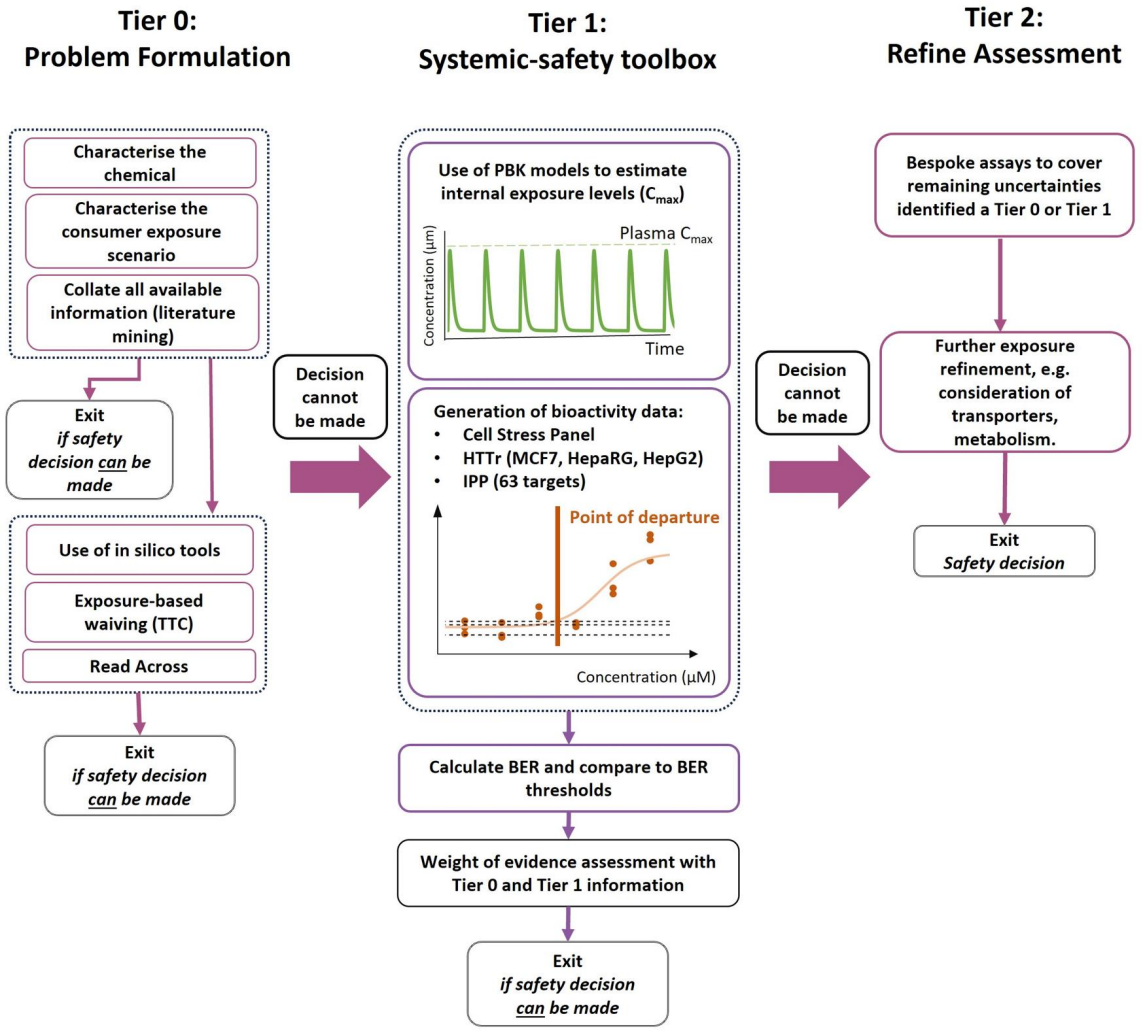
A risk assessment framework inspired by those of the Seurat-1 project, the EPA blueprint, and previous NGRA case studies (Berggren et al. 2017; Thomas et al. 2019; Baltazar et al. 2020; OECD 2021) showing where this systemic-safety toolbox could sit in an early tier data generation phase following collation and appraisal of all existing information at Tier 0. At each potential “exit” the outcome can be a safety decision of low-risk or uncertain risk. Cases where the risk is uncertain can potentially be progressed through to higher tier testing if this can address any remaining uncertainties identified at earlier tiers, e.g. mechanism of action-based testing. The use of the systemic-safety toolbox at Tier 1 is intended to address cases where there are data gaps at Tier 0 specifically regarding systemic toxicity.

The purpose of the present study was to assess whether these threshold BERs remain protective and useful for a larger set of chemicals and exposures. The selection of these chemicals was performed in a semirandomized way to obtain a diverse and representative set of test chemicals in a manner that avoided, as much as possible, potential biases, such as skewing toward highly active or completely inert chemicals. A total of 38 chemicals and 70 associated exposure scenarios were selected for data generation, covering a variety of use-case categories, multiple routes of exposure, and both high- and low-risk classifications.

Previously published evaluations of NAMs have indicated that PoDs derived from in vitro assays tend to be more conservative compared with those obtained from traditional animal studies (Paul-Friedman et al. 2020; Reardon et al. 2023; Zobl et al. 2024) or historical published human PoDs (Reinke et al. 2024—submitted). Consequently, in addition to generating the previously established performance metrics (protectiveness and utility), a secondary objective of this work was to evaluate the conservatism of the decisions made using the systemic-safety toolbox. This was done by comparing the lowest PoD across the bioactivity platforms (PoD_NAM_) after performing reverse dosimetry with the lowest PoD from available repeat dose in vivo animal studies (PoD_traditional_) for a select group of chemicals, where a robust in vivo PoD could be determined.

## Materials and methods

### Materials

Test chemicals were purchased from Sigma, Sigma-Supelco, TCI, Carbosynth, Cambridge Bioscience, Abcam, Molport, and LGC. The same batch of chemical was tested across all the bioactivity assays mentioned below (details in Table S6).

### Methods

#### Chemical selection

**Fig. 2.**
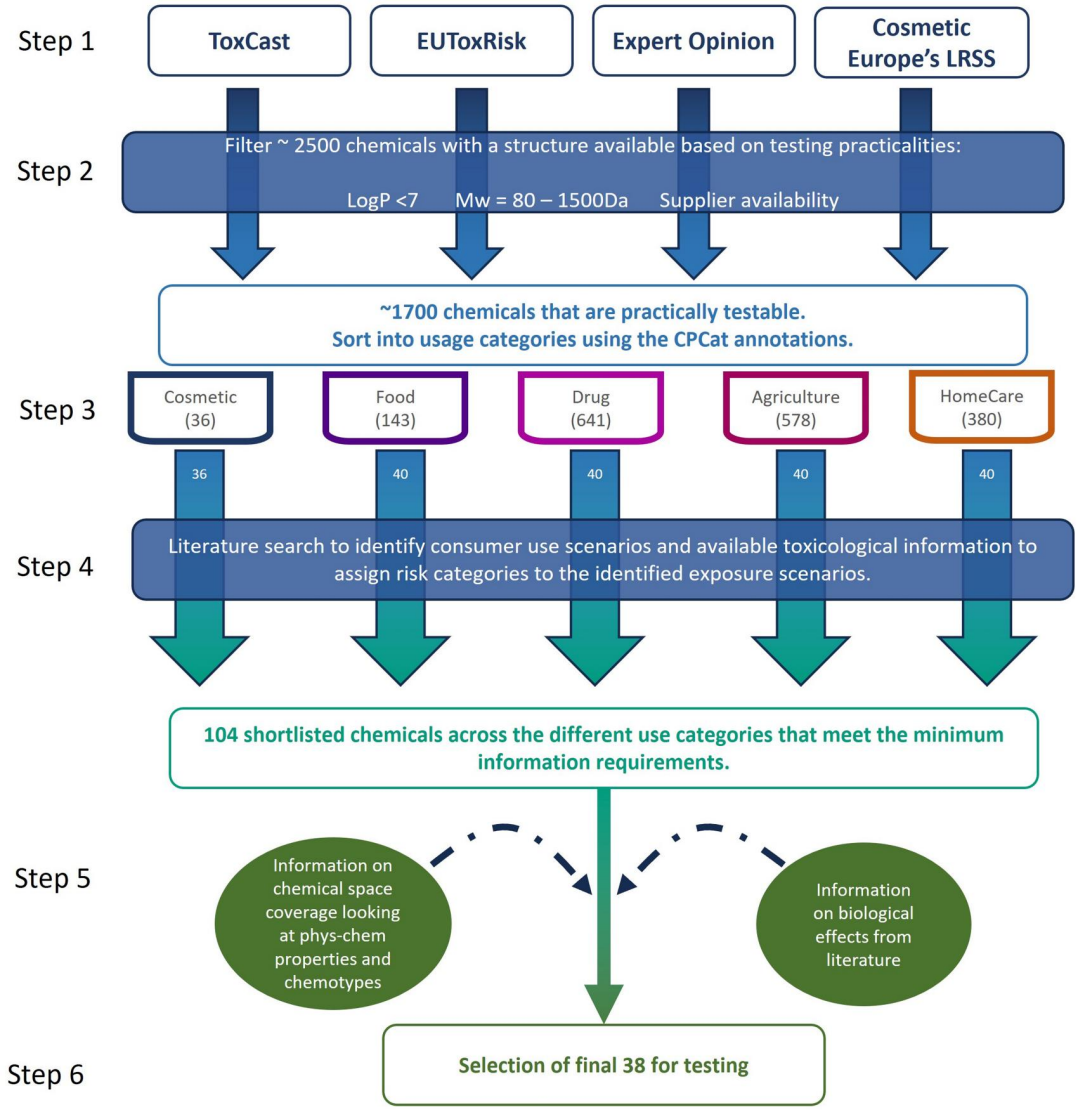
Overview and flow of the chemical selection process as described in Table S1 resulting in 38 chemicals to progress to data generation for evaluation of the systemic toolbox and workflow.

The chemical selection process was divided into 6 steps (listed in Table S1 and shown in Fig. 2). An initial list of over 2,000 potential chemicals was compiled from various databases, published reference test chemical lists and expert opinion with the aim of spanning a broad range of chemistries, exposures, and biological effects while also representing chemicals that are in use currently across different sectors. Chemicals were then removed from the list for any of the following reasons a supplier for the chemical could not be identified; the chemical had a molecular weight greater than 1,500 Da, and therefore would have negligible systemic bioavailability; the properties of the chemical would likely make it incompatible with the high-throughput nature of the assays used in the systemic-safety toolbox, e.g. due to being volatile or semivolatile. The final set of chemicals was then selected using stratified sampling based on different predefined use-case categories which is an unbiased, i.e. random, selection according to the different category groups. First, the list of chemicals obtained at step 2 was separated into these 5 categories based on the first 20 use annotations assigned in the Chemical and Product Categories database (CPCat, https://actor.epa.gov/cpcat/faces/home.xhtml). These categories were: Agricultural, Drug, Homecare, Food, Cosmetic. Forty chemicals were then randomly selected from each category and then taken forward to form a shortlist, although only 36 chemicals were found to have a cosmetic use listed, giving a total of 196 compounds. A literature search was conducted to see if exposure scenarios could be identified for which data existed to assign a risk category. Any chemicals where this was not possible were removed from the list. Where multiple chemicals of the same class were shortlisted only one was selected to maximize the coverage provided by the test chemicals; e.g. 4 organophosphate compounds were shortlisted of which only chlorpyrifos was selected for testing based on the availability of existing toxicological information for assigning a risk classification. The final selection of 38 test chemicals was then carried out ensuring adequate coverage of the chemical space and diversity of modes of action and toxicities identified from literature searching.

#### Assignment of risk classifications to benchmark chemical-exposure scenarios

Following the selection process outlined above, at least one human exposure scenario per chemical was identified from either typical consumption levels, treatment regimens for medicines, literature case studies, cosmetic regulatory limits, or occupational limits. Regulatory bodies such as the European Food Safety Agency (EFSA), European Scientific Committee on Consumer Safety (SCCS), U.S. Environmental Protection Agency (EPA), and the U.S. Food and Drug Administration (FDA) provide comprehensive reviews of chemicals used in food, pesticide, and consumer product scenarios. Separately, a literature search was performed to identify any case reports or literature reviews demonstrating evidence to substantiate the assignment of either a high- or low-risk classification. These data, and conclusions from the regulatory opinions where available, are documented in Table S1. Two of the test chemicals had both low- and high-risk exposures from a systemic perspective: ketoconazole and trimellitic anhydride.

#### Computing chemical space

The chemical space was investigated in 2 ways. First, to aid chemical selection process, structures of the 104 chemicals obtained at step 5 (Fig. 2) were represented by their physico-chemical properties (logP, WS, VP, BP, MP, Henry constant, logKoa) calculated using OPERA tool (Mansouri et al. 2018) and chemotypes calculated by Toxprint (Yang et al. 2015). Following this, the chemical space was visualized using principal component analysis (PCA) within the JMP software (JMP, Version 14.1.0 SAS Institute Inc., Cary, NC, United States, 1989–2023). A more in-depth analysis of the chemical space was then performed after step 6 (once the final selection had been made), where each structure was represented using RDKit descriptors (library version: 2023.03.2, python version: 3.11.14), fingerprints, and chemotypes. The composition of the test chemical list was compared with the initial starting selection of 2,366 chemicals compiled from various sources with cross-sector uses (listed in Table S1), and then also compared with a reference database of 1,613 chemicals used in the cosmetics sector, as defined by the usage category annotations in CPCat database (on January 14, 2020). The 2D chemical structures for all 38 test chemicals were generated from SMILES (Simplified Molecular Input Line Entry System), although butylated hydroxyanisole is a mixture of isomers and has been represented by 2 structures. Duplicates were removed from all 3 datasets leaving a total of 39 test chemical structures, 2,331 structures from the initial list, and 1,408 CPCat cosmetic structures. Mixtures were not removed, and chemicals were not desalted to better represent the true chemistries, instead of differentiating salts of the same substance for example. An algorithm was developed to compute chemical space, where all chemicals were represented by molecular descriptors computed using the python library RDKit and structural fingerprints generated using Chemotyper (version 1.0). The dataset then underwent a first reduction stage through active removal of descriptors if the maximum tolerated cross-correlation (defined as Pearson’s *r*^2^) and minimum accepted diversity criteria were not met (0.8 and 0.3, respectively), full details of this stage can be found in Supplementary 2. A second reduction stage was then performed via PCA to the number of components needed to explain a desired amount of variance. The final reduction stage was carried out using the t-distributed Stochastic Neighbour Embedding (t-SNE) technique to project the dataset onto 2 dimensions and to visualize it graphically.

#### Estimating systemic exposures using PBK modelling

Internal exposure estimates were generated using PBK models developed using Gastroplus 9.8. (Simulation Plus, Lancaster, CA, United States), a specialist software that incorporates modules capable of simulating different routes of exposure including oral, dermal, and IV. Models are parameterized using data where applicable across diverse parameters including logP (octanol-water partition coefficient), water solubility, unbound fraction in plasma (*f*_up_), blood:plasma ratio (Rbp), hepatic intrinsic clearance (Cl_int_), Madin–Darby Canine Kidney permeability (*P*_app_), pKa (acid dissociation constant), and intestinal absorption or skin penetration where applicable. Each chemical exposure scenario was modelled following a tiered framework outlined in Moxon et al. (2020), separated into different levels of complexity and refinement based on the input parameters:

- *Level 1 (L1)—in silico only*: Chemical specific parameters are derived solely from ADMET predictor in silico
- *Level 2 (L2)—in silico and in vitro*: At least one of the specified parameters is obtained from in vitro measurements, whereas the remaining parameters remain derived from in silico predictions.
- *Level 3 (L3)—in silico, in vitro, and clinical data*: Similar to L2, chemical specific parameters are derived from in vitro measurements, where available. Clinical data are then utilized to calibrate the model through refinement of key parameters identified through a combination of sensitivity analysis and expert judgement. Notably, the clinical data used for calibration are from exposure scenarios distinct to those being modelled, and therefore, the L3 *C*_max_ predictions are not measured values.

#### Bioactivity assays

##### Concentration range setting

A cytotoxicity assessment using cellular ATP and LDH release measurements was performed for all compounds using the cell lines tested in the cell stress (HepG2) and HTTr (HepG2, HepaRG, and MCF7) platforms (unpublished data). The same concentration setting procedure was used for the cell stress and transcriptomics platforms as previously described (Middleton et al. 2022), where the maximum dose tested was chosen based on the minimum of either a compound’s solubility limit or the concentration at which cytotoxicity was observed. The cytotoxicity data were also used to select the concentrations used for each compound tested in the IPP screen (see below).

##### Cell stress panel

Compounds were tested using the previously developed cell stress panel (Hatherell et al. 2020) and the expanded biomarker panel outlined in Middleton et al. HepG2 cells were treated with all compounds for 24 h across 8 concentrations prior to biomarker analysis. The same plate layout and number of replicates (3 biological and 2 technical) were used as previously for each assay within the panel.

##### High-throughput transcriptomics

HepG2, HepaRG, and MCF7 cells were treated with each compound for 24 h across a dose range of 7 concentrations and lysed using TempO-Seq lysis buffer (BioSpyder Technologies, proprietary kit, see Middleton et al. 2022 for method details). Sequencing was performed using TempO-Seq (BioClavis) version 2 of the human whole transcriptome panel and analyzed as described previously in Middleton et al.

##### In vitro pharmacological profiling

The IPP platform used here contains 63 targets with known safety liabilities that were tested in binding, enzymatic, coactivator recruitment, and luciferase assays; a full list of which is available in Table S3b. Forty-four of the targets in the IPP panel have been associated with in vivo adverse drug reactions by the pharmaceutical industry (Bowes et al. 2012). To expand biological coverage into additional targets implicated in developmental toxicity, a further 19 targets were added to the panel based on a literature search (National Research Council Committee on Developmental Toxicology 2000; Wu et al. 2013; Rajagopal et al. 2022). Three targets tested in Middleton et al. were no longer available, mineralocorticoid receptor NR3C2 and retinoic acid receptor gamma (RARG); the aryl hydrocarbon receptor assay was also no longer available, however, AhR is an endpoint measured in the cell stress panel. A PoD from the IPP panel is derived in 2 stages: Screening and dose response. In the first stage, the screening is done in all assays at a fixed concentration of the substance (2 replicates), at 10 or 100 µM, based on the previous cytotoxicity measurement in HepG2, HepaRG, and MCF7 cells. Targets showing an inhibition or stimulation higher than 50% are followed up using concentration-response assays from which an IPP-specific PoD is determined. For more information refer to Middleton et al.

#### Data analysis

##### PoD estimation

PoDs were calculated for each bioactivity platform using the same methods and settings as described in Middleton et al. (2022). For the IPP platform, either the EC_50_ (the concentration of test chemical at which the response is half maximal) and IC_50_ (the concentration of test chemical at which the response is half maximal against the control agonist) was calculated where appropriate. For the HTTr, 2 methods were used to analyze the gene expression data: BMDExpress2 (Phillips et al. 2019) and BIFROST (Reynolds et al. 2020). BMDExpress2 was used to estimate the Benchmark concentration (BMD) values for each probe as well as corresponding upper and lower confidence internal bounds (BMDU, BMDL), based on a Benchmark response (BMR) factor of 10%. Pathway enrichment was performed using BMDExpress2 and Reactome pathways. The mean pathway BMDL was calculated for each pathway that was determined to be significantly enriched by taking the mean BMDL value across all significant probe level BMDLs (see Middleton et al. for further details). BIFROST was used to calculate the “global PoD,” which corresponds to the minimum effect concentration across all genes for a given test substance (see Reynolds et al. 2020; Middleton et al. 2022 for further details). A version of BIFROST was also used to analyze the cell stress panel concentration response data and used to estimate a CSP global PoD per chemical (see Hatherell et al. 2020; Middleton et al. 2022 for further details).

##### Calculation of the BER

BERs were computed using the same approach as defined in Middleton et al. (2022). In brief, the BER is taken to be the ratio between the minimum PoD and the maximum total plasma concentration (*C*_max_), estimated using Gastroplus at either parameterization level L1, L2, or L3. The set of bioactivity platform PoDs from where the minimum was taken are:

- The minimum IC50 or AC50 from the IPP panel (if available).
- The CSP global PoD, as estimated using BIFROST
- The minimum pathway BMDL from the transcriptomics platform (one for each cell line), estimated using BMDExpress
- The global PoD from the transcriptomics platform (one for each cell line), estimated using BIFROST.

Thus, for a given chemical-exposure scenario, one BER estimate is calculated for each *C*_max_ estimate corresponding with each PBK parameterization level.

##### BER decision thresholds

In the systemic-safety toolbox, the BER is used as a threshold to decide whether an exposure scenario is low-risk or uncertain risk. The decision thresholds used in this work were the same as established in Middleton et al. (2022). Here, there is one threshold value for each PBK level, reproduced in Table 1. The threshold values were calculated for each PBK parameterization level, based on a Bayesian statistical model of the PBK prediction errors from a set of 8 benchmark exposure scenarios published in Middleton et al.

**Table 1.** Reproduced from Middleton et al., a summary of the BER thresholds in the prototype decision model required at each PBK level to conclude low-risk.

PBK parameterization level	BER threshold
L1 (in silico parameters)	110
L2 (at least 1 in vitro parameter)	11
L3 (model calibrated to human clinical data)	2.5

##### Protectiveness and utility metrics

The protectiveness and utility metrics as defined in Middleton et al. were used to assess the overall performance of the toolbox and workflow. Protectiveness corresponds to the proportion of high-risk benchmark scenarios correctly identified as not low-risk (i.e. as uncertain risk) and is calculated as $\frac{H_{U}}{H_{U}+H_{L}}$, where $H_{U}$ is the number of high-risk exposures identified as uncertain risk, $H_{L}$ is the number of high-risk exposures identified as low-risk. Utility corresponds to the proportion of low-risk scenarios correctly identified as such, and is given by $\frac{L_{L}}{L_{L}+L_{U}}$, where $L_{L}$ is the number of benchmark low-risk exposures correctly identified as low-risk and $L_{U}$ is the number of benchmark low-risk exposures identified as uncertain risk.

#### Comparison to in vivo toxicological data

To further evaluate the performance of these selected NAMs in the context of early-tier decision-making, a comparison to the performance of in vivo data used in the same manner was performed and protectiveness and utility metrics calculated. To do this, in vivo data were identified from 3 sources: ToxRefDB (version 2.1, https://www.epa.gov/comptox-tools/downloadable-computational-toxicology-data), the supplementary material of Paul-Friedman et al. (2020) and published regulatory opinions. Repeat-dose data were identified for 25 of the test chemicals and the *minimum* NOAEL or NOEL for each from any repeat-dose study are summarized in Table S5. To enable a comparison of these in vivo PoD to the NAM PoD reverse dosimetry was performed using the highest available *C*_max_ estimated for each chemical exposure scenario and converting the NAM PoDs from µM concentrations to equivalent external applied doses in mg/kg bw/d to allow comparison to in vivo PoDs recorded as external doses, see Supplementary 2 for details of the calculation. A threshold MoS of 100 has been used to allow calculation of protectiveness and utility metrics, as this is widely regarded as a starting point that accounts for various uncertainty factors and represents a safe consumer exposure (SCCS 2023).

### Data repository

Raw experimental data from all assays, together with detailed reports for the HTTr and CSP analysis, and a summary of the PBK model predictions, are provided through the Dryad repository, available at: https://doi.org/10.5061/dryad.v6wwpzh57.

## Results

### Coverage of the toolbox benchmark chemical-exposure scenarios

The chemical selection process outlined in Fig. 2 and Table S1 resulted in a list of 38 chemicals, with 70 exposure scenarios identified (46 high-risk and 24 low-risk). Among these were 9 chemicals primarily associated with cosmetic use, 21 primarily associated with medicinal use, 3 associated with food exposures, 5 agricultural chemicals, and 1 primarily associated with occupational use (Table S1). As exposure is an integral part of this workflow, coverage can also be viewed in terms of the exposure scenarios identified and modelled for the selected chemicals. Overall, 2 exposures were via the inhalation route, 6 via intravenous administration, 13 via dermal application, and 49 via oral administration. As described earlier, the different PBK levels rely on different types of input parameter; of the scenarios modelled, 2 of the chemicals had only in silico parameters available and so it was only possible to calculate L1 *C*_max_ estimates. These were 1,2-octanediol and panthenol, all other chemicals had in vitro data for at least one input parameter to allow L2 predictions to be made. Clinical data were available for calibration of 47 PBK models, giving an L3 prediction; however, all but 3 of these were drugs used within the therapeutic window and classified as high-risk (from a consumer good perspective). The 3 models for low-risk benchmark scenarios able to be calibrated with clinical data, were the dietary consumption of butylated hydroxyanisole and the short- and long-term use of shampoos containing ketoconazole to treat dandruff.

We sought to map the diversity of the chemical and biological space provided by the choice of test chemicals. Table S1 summarizes the risk classifications and some of the literature used to investigate the biological coverage of the test chemicals through the effects reported in humans, either on target in the case of some of the drugs, or adverse effects. The test chemical set contains many that are not associated with any adverse effects, some with local site of administration effects and some associated with nonspecific and specific toxicities. Of the examples where evidence of effects in humans was found, these spanned hepatoxicity events, immune system effects, blood-based disorders, nervous system disruption, neurological effects, cardiac effects, nephrotoxicity, gastrointestinal issues, and an example of pulmonary fibrosis. In some cases, the mechanism of action is also the mechanism of toxicity, such as digoxin where the mechanism of action is inhibition of the Na+/K-ATPase enzyme (Krstic et al. 2003) which is associated with cardiotoxicity. This is also the case for warfarin where the target enzyme in the blood and resulting change in blood coagulation ability is the desired activity but requires regular monitoring to avoid life-threatening major bleeds; and for ibuprofen, where its anti-inflammatory effects are exerted through inhibition of COX-1 and COX-2, with excessive inhibition leading to the documented adverse gastric effects among other effects (Robinson 1983).

**Fig. 3.**
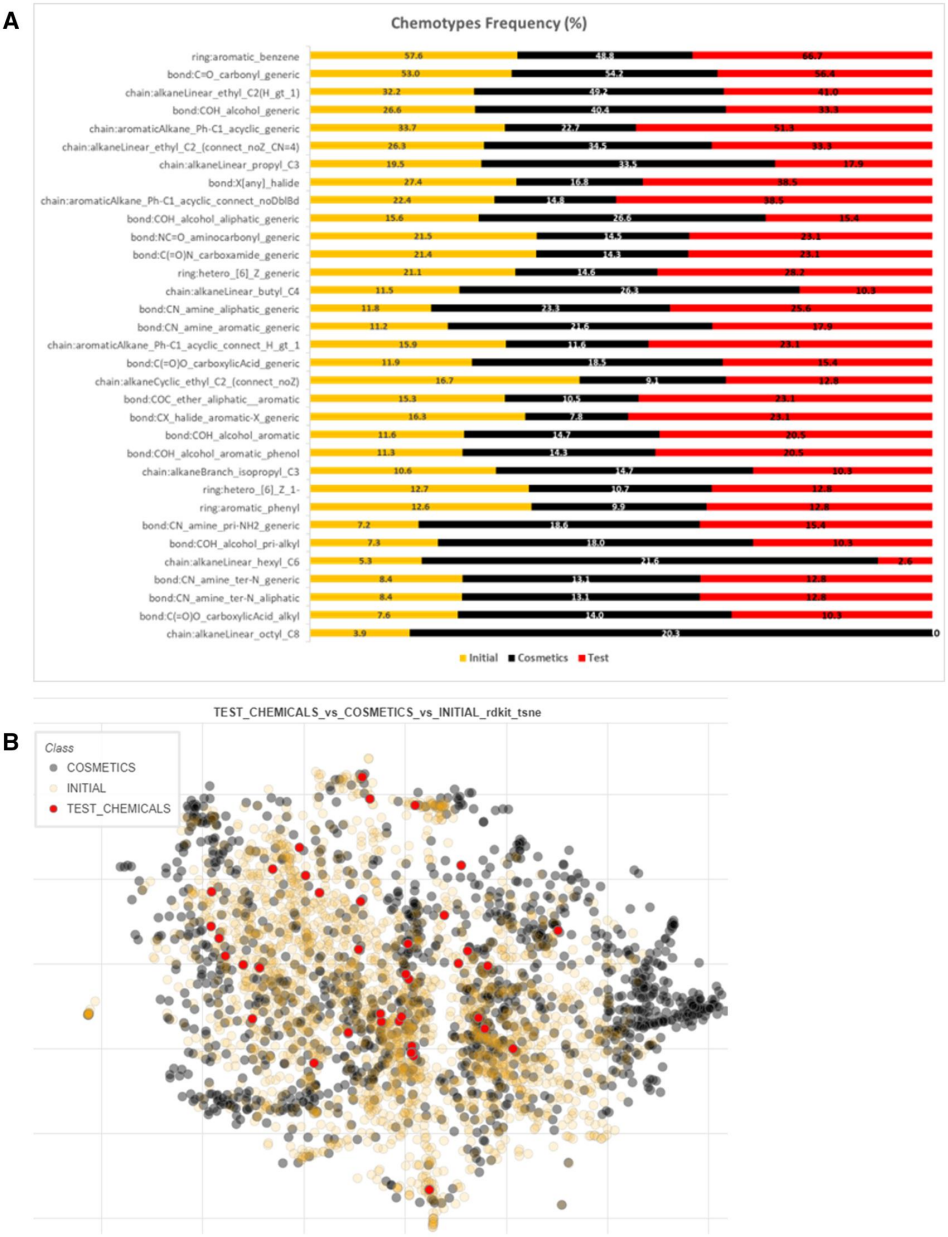
Two visualizations of the chemical space covered by the 3 different chemical sets analyzed: yellow represents the initial collated list of ∼2,500 chemicals in step 1 of the selection process; black represents the chemicals annotated in the CPCat database as used in cosmetics; and red represents the 38 chemicals tested in this work. (A) Histogram of the 33 most frequent chemotypes present within the 3 chemical lists. Most chemotypes show an even representation, apart from linear alkane chains that are comparatively underrepresented in the test chemical list. (B) t-SNE visualization of the chemical space covered by the different chemical lists. The region on the right-hand side with no representation in the test chemical set, largely represents chemicals with long alkane chains which corresponds with the chemotype frequency analysis.

In terms of coverage of different chemistries, the selection process outlined in Fig. 2 and Table S1 partially defined the chemical space covered in terms of logP and molecular weight. Of the chemicals that were in the final selection, molecular weights ranged from 100.12 to 780.9 g/mol and the predicted S logP ranged from −2.32 to 5.99. In addition to using the physico-chemical descriptors of each chemical, the ToxPrint chemotypes were used to examine the chemical space. These are structural fragments encoded for connectivity (which may extend beyond a single connected fragment) and also, when desirable, for physico-chemical properties of atoms, bonds, fragments, electron systems, and even a whole molecule (Yang et al. 2015). A set of reference chemicals were used to assess the diversity of the 38 test chemicals. These reference chemicals were obtained from 2 different lists, the initial list of ∼2,500 structures compiled in Step 1 of the selection process and an extraction of chemicals listed as used in cosmetics with the CPCat database as an exemplar of chemicals that a significant portion of the public are exposed to. From the entire repository of the ChemoTyper tool of 729 chemotypes, 572 chemotypes have been identified across all 3 sets of chemicals; 525 chemotypes in the initial set, 462 chemotypes in the cosmetics set, and 181 chemotypes in the test chemicals. Figure 3A shows the 33 most frequent chemotypes (frequency calculated as percentage) present in each set of chemicals. The first 2 most frequent chemotypes (∼ or above 50%) for all 3 sets of chemicals are: ring:aromatic_benzene and bond:C=O_carbonyl_generic. In general, there appeared to be good concordance between the chemical sets regarding the proportion of chemotypes present in each with notable exceptions relating to the chemotypes describing longer (C8 and above) alkyl linear moieties, widely present especially in cosmetics set, being absent in the 38 test chemicals.

In addition to using the Toxprint chemotypes, the chemical space was visualized through representation by RdKit descriptors using the t-SNE technique (see Methods section) of our test chemicals, initial list, and the CPCat cosmetics (overall 3,778 chemicals). As is shown in Fig. 3, the 38 test chemicals are scattered over much of the chemical space represented by compounds from initial list and cosmetics. However, there are structural regions not represented by test chemicals, such as structures with long aliphatic chains (right side of the Fig. 3B, mainly populated by cosmetics) which agrees with the chemotype frequency analysis presented in Fig. 3A. Other examples of structural features that are missing in the test chemicals are aromatic polyamines, sulphonated polyaromatics, perfluorinated, and azo moieties. Overall, the 38 test substances are structurally diverse considering the small size of this sample.

### Performance of the toolbox in terms of protectiveness and utility metrics

To investigate whether the systemic safety toolbox is fit-for-purpose, in the context of early tier safety decision-making, the same metrics were used as described in the initial pilot study (see Middleton et al.). These were protectiveness (how many of the high-risk scenarios does the decision model classify correctly as “uncertain risk”; therefore requiring further assessment) and utility (how many of the low-risk scenarios does the decision model correctly classify as low-risk). In theory, the optimal toolbox would have maximal (100%) protectiveness and utility and ensure that high-risk exposures are not incorrectly identified as low-risk. In other words, the toolbox would enable safety decisions to be made, that would protect people against all high-risk exposure scenarios. However, many chemicals that are considered safe in normal use will induce bioactivity at relevant exposure levels and therefore be identified as uncertain risk using the first-tier toolbox. Obtaining 100% utility at this tier is not the primary intention and is therefore not expected, however, it is envisaged that when using the toolbox as part of a tiered approach (as shown in Fig. 1) truly low-risk exposures that are identified as uncertain risk, could be correctly identified as low-risk using further higher tier tools. Further testing can also be used to increase the certainty in cases where truly high-risk exposures are identified as uncertain risk using the toolbox.

**Fig. 4.**
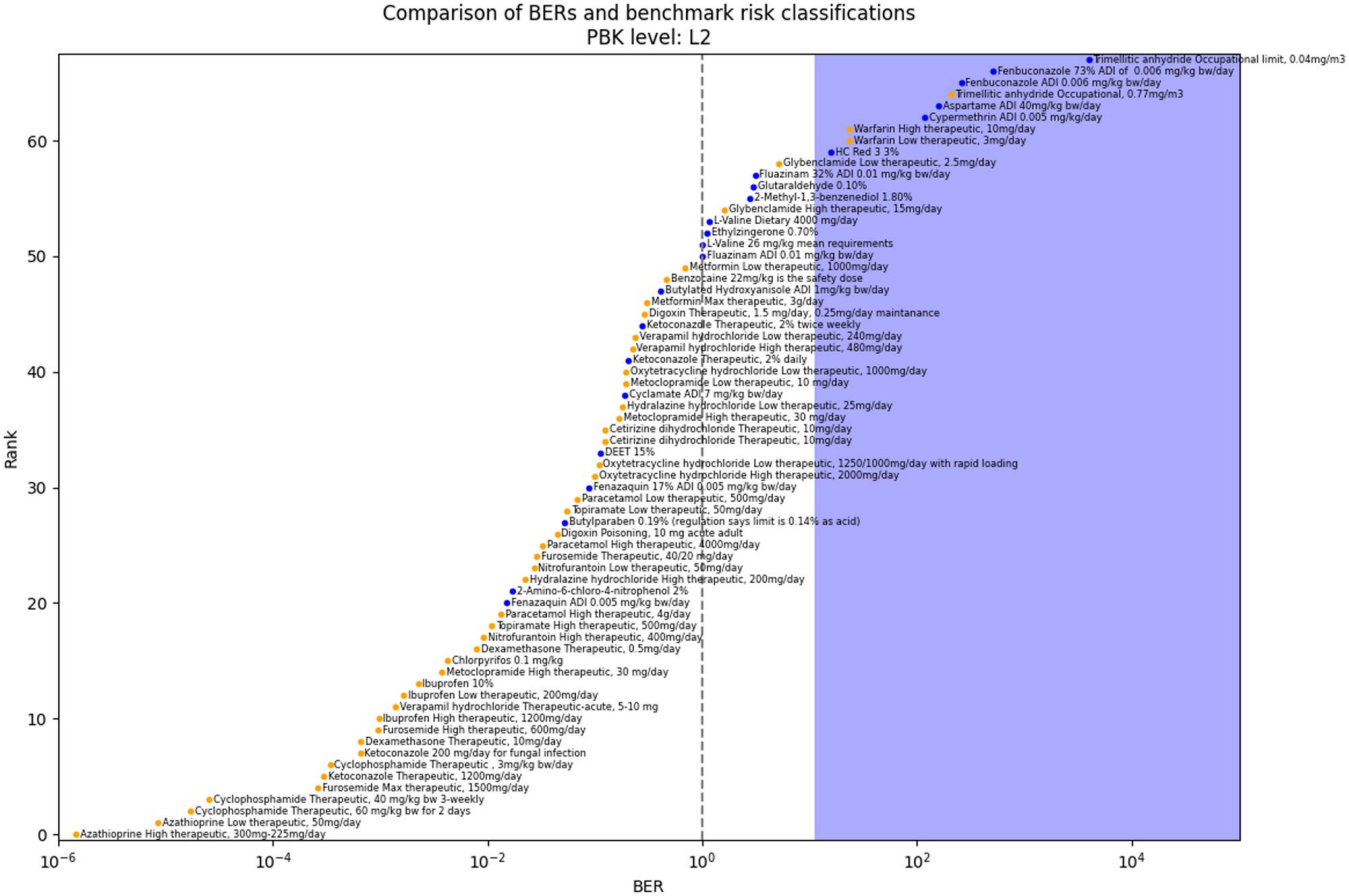
Plot showing BER values for 68 chemical exposure scenarios where L2 exposure estimates were available. Yellow dots represent chemical exposure scenarios classified as high-risk, blue dots represent chemical exposure scenarios classified as low-risk. The blue-shaded region represents a BER of 11 or above, with a BER >11 being the previously determined threshold for a low-risk decision at L2. The black dashed line represents BER = 1. N.B. The scenarios with only L1 *C*_max_ predictions available are not included in this, i.e. 1,2-octanediol in a body lotion and panthenol in a body lotion, BER plots for L1 exposure estimates and L3 only can be found in Supplementary 2 Figs S2–S4.

BERs were calculated using the lowest PoD across all bioactivity platforms for each chemical and dividing them by the plasma *C*_max_ estimates for each chemical exposure scenario. Figure 4 shows the L2 BER point estimates calculated in this evaluation. These BER estimates were then compared with the thresholds identified in the previous work and summarized in Table 1, with the blue region in this figure indicating when a BER exceeds the associated decision threshold for identifying a low-risk scenario using the decision model. Chemical exposure scenarios within the white region represent cases, where the BER is below this threshold and would be classified by the model as uncertain risk (therefore needing further evaluation). At L2, with PBK models parameterized with in vitro data, the decision model identified 43/46 high-risk exposure scenarios as uncertain risk, and 6/22 low-risk exposure scenarios as low-risk; giving a protectiveness of 93% and utility of 27%. Only trimellitic anhydride and warfarin had high-risk exposure scenarios misclassified as low-risk at this stage. These metrics are improved if you also consider situations where better data are available to parameterize the PBK models and therefore there is less uncertainty surrounding the resulting exposure estimate. In these cases, where human clinical data can be used to calibrate the PBK models and an L3 *C*_max_ prediction is available, the protectiveness increased to 98% overall. With the reclassification of the upper bound of the therapeutic window for warfarin as uncertain risk instead of low-risk due to the refined PBK model. Although not all chemicals have clinical data available which means that at L3 there are only 41 high-risk exposure scenarios and 3 low-risk exposure scenarios instead of 46 and 24, respectively. Although PBK models parameterized with in vitro data, i.e. L2, are the most likely future scenario for a novel chemical risk assessment, the same analysis of the toolbox performance can be done using L1 and L3 inputs as summarized in Table 2. For example, if only L1 PBK *C*_max_ estimates are used, with a BER threshold of 110, then the model performance was 93% protectiveness and 8% utility. In Table 2 below, the “highest” PBK level takes the best available exposure estimate across all predictions and compares it with the relevant thresholds; i.e. where only L1 exists the BER must >110, where an L2 prediction exists this is used instead and the BER must >11, and where an L3 prediction exists this is used and the BER >3. When the highest PBK level is used only the high-risk trimellitic anhydride scenario, and the low therapeutic dose of warfarin are misclassified as low-risk.

**Table 2.** Protectiveness and utility statistics for the toolbox varies depending on the parameterization of the PBK model used.

PBK level	Protectiveness	Utility
L1	93% (43 out of 46)	8% (2 out of 24)
L2	93% (43 out of 46)	27% (6 out of 22)
L3	98% (40 out of 41)	0% (0 out of 3)
Highest	96% (44 out of 46)	29% (7 out of 24)

The availability of in vitro and clinical data means that the number of benchmarks at each PBK level changes. The “highest” available PBK level statistics consider all available models and compare with appropriate benchmarks to derive the performance statistics, i.e. where L1 *C*_max_ available the BER threshold of 110 is used, but for L2 and L3, the thresholds of 11 and 2.5, respectively, are used.

There are a total of 8 different PoD types generated by the systemic-safety toolbox: 1 associated with IPP, 1 with CSP, and 2 for each of the 3 HTTr cell lines that were tested (one based on the global PoD obtained using BIFROST and one on the minimum pathway BMDL obtained using BMDExpress2.0). The workflow adopted in this work (see Middleton et al.) used the minimum overall PoD for a given chemical to calculate a BER. Across the different exposure scenarios considered in this work (see Table S2c), IPP gave the lowest PoD for 11 chemicals, CSP gave the lowest PoD for 5 chemicals and HTTr (the global PoD obtained using BIFROST) gave the lowest PoD for 25 chemicals (8 in HepaRG, 6 in HepG2, 11 in MCF-7). It is expected that the pathway level PoD from the HTTr data will always be higher than the gene level PoD derived from the BIFROST analysis, as a pathway level PoD relies on the differential expression of multiple genes within the same pathway. As previously observed in Middleton et al., none of the chemicals tested had the minimum pathway BMDL as the leading PoD, so from here on any reference to a HTTr PoD refers to the BIFROST gene level PoD and not the pathway BMDL, unless stated otherwise. Table 3 below summarizes the protectiveness and utility when calculating the BER using different bioactivity platform combinations, together with the highest available PBK *C*_max_ estimate. The bioactivity platform that provided the greatest protectiveness, when used in isolation, was the HTTr (89%), with a utility of 33%. The maximum achievable protectiveness of 96% came from combining the IPP and HTTr PoDs; the addition of CSP PoDs did not increase this value. The PoDs calculated from the cell stress panel are typically in similar ranges to the PoDs calculated from one or more of the cell lines tested in the HTTr, and therefore, from this analysis, incorporation of the cell stress panel does not change the performance of the toolbox in terms of identifying low- and high-risk chemical exposures. Nevertheless, of the examples where the HTTr is required to make a protective decision, there are 6 chemicals that rely solely on the HTTr PoDs and 15 examples where the PoD from the HTTr data is determined based on isolated changes in either 1 or 2 genes identified as differentially expressed but at concentrations an order of magnitude or more below other responding genes. For example, these isolated gene changes were often seen in metabolism related genes, and specifically CYP1A1, which does not necessarily indicate an adverse effect, more a cellular response to xenobiotic exposure.

**Table 3.** Performance of the toolbox when different combinations of the bioactivity platforms are used for decision-making.

IPP	CSP	BIFROST HTTr global	HTTr—BMD minimum pathway	Protectiveness	Utility
Y	Y	Y	Y	96% (44 out of 46)	29% (7 out of 24)
Y				86%^a^ (25 out of 29)	76%^a^ (13 out of 17)
	Y			61% (28 out of 46)	62% (15 out of 24)
		Y		89% (41 out of 46)	33% (8 out of 24)
			Y	48% (22 out of 46)	62% (15 out of 24)
Y	Y			83% (38 out of 46)	54% (13 out of 24)
Y		Y		96% (44 out of 46)	29% (7 out of 24)
Y			Y	74% (34 out of 46)	54% (13 out of 24)
Y	Y	Y		96% (44 out of 46)	29% (7 out of 24)
Y	Y		Y	83% (38 out of 46)	54% (13 out of 24)
	Y	Y		89% (41 out of 46)	33% (8 out of 24)
	Y		Y	65% (30 out of 46)	62% (15 out of 24)
	Y	Y	Y	89% (41 out of 46)	33% (8 out of 24)
		Y	Y	89% (41 out of 46)	33% (8 out of 24)
Y		Y	Y	96% (44 out of 46)	29% (7 out of 24)

a Only a subset of chemicals have a PoD calculated from the IPP assay as can be seen in the total number of scenarios addressed using the IPP results. Chemicals that did not trigger a response over the threshold in the screening phase did not have a PoD calculated.

### Identifying gaps in the biological coverage of the toolbox

The results of the bioactivity data generated were compared with known literature for the test chemicals, and each chemical exposure scenario was grouped into one of the following categories: (i) the systemic-safety toolbox is not protective (*n* = 2); (ii) the toolbox is protective but the mechanism of action is not detected (*n* = 9); (iii) low-risk chemical exposure scenarios are classified as requiring further assessment (*n* = 15); (iv) the correct classification is made/the mechanism of action is detected (*n* = 13). The predicted *C*_max_ was above the minimum PoD for all but 2 of the chemicals that had a high-risk exposure associated; the 2 exceptions were for trimellitic anhydride (a case report where the exposure exceeds the occupational exposure limit) and warfarin (for 2 different therapeutic doses).

The benchmark risk classification for trimellitic anhydride as high-risk based on traditional data is due to the observations that serum IgE and other immune markers were elevated in humans secondary to respiratory effects. For this compound, the reactivity predictions combined with the known inhalation route of exposure would most likely have led to a custom testing strategy with the primary concern being respiratory sensitization (Sadekar et al. 2021). The toolbox is intended to focus on the systemic toxicity risk and should not be considered in isolation of addressing other endpoints with other techniques, and it is expected that the sensitizing nature of trimellitic anhydride would be the limiting factor in the assessment of this occupational exposure. The other case where the toolbox is not protective is for warfarin, which is known to have a very specific effect through inhibition of the vitamin K epoxide reductase enzyme (VKORC1) and subsequent impact on the anticoagulation cascade. The PoDs for warfarin in this evaluation ranged from 13.6 µM (HepaRG BIFROST global PoD) to 259 µM (HepaRG minimum pathway BMDL), which were markedly higher than the typical *C*_max_ reported in the literature (0.6–6 µM). Literature data on the activity of warfarin on the VKORC1 enzyme show IC50 values ranging from 24.7 nM to 4 µM (Bevans et al. 2013), which, if incorporated into a BER calculation would give a BER≪1 and move both warfarin exposures to an uncertain risk conclusion at all PBK levels, however, an assay of this nature was not included in our toolbox. This is a specific mechanism of action but demonstrates the importance of combining data from specific and nonspecific in vitro assays as lines of evidence in an early tier systemic-safety toolbox.

Of the 10/38 examples where the known mechanism of action was not detected (see Table 4 for details), a protective decision was still able to be made for the high-risk exposure scenarios associated with 9 of these, i.e. all but warfarin. In some cases, the mechanism of action was not detected because the target of the chemical was not present in the bioactivity platforms comprising the toolbox. Benzocaine forms methaemoglobin which is not a biomarker measured within any assay; hydralazine hydrochloride and glibenclamide both work through affecting movement of ions, however, the current panel of assays does not contain the relevant targets (Ellershaw and Gurney 2001; Hartman et al. 2016; Palacio et al. 2021). Nevertheless, protective BERs for all were determined from the HTTr data. Another chemical with a specific mechanism of action is topiramate which primarily acts as a GABA-receptor antagonist, however, this was not detected in the IPP due to the specificity of this chemical for a subunit of the receptor not present within the panel. Inhibition of the carbonic anhydrase II enzyme led to the determination of a BER<L2 threshold, for which there is evidence in the literature of this interaction (Sano et al. 2021; Pearl et al. 2023). In some other cases the mechanism of action is driven by formation of a metabolite; 3 examples of this are chlorpyrifos, cyclophosphamide, and azathioprine. The metabolism of cyclophosphamide and azathioprine lead to metabolites that interfere with DNA (Chrzanowska et al. 1985; Highley et al. 2022), and while this was not detected in the biomarkers measured in the CSP, both compounds were highly active across all bioactivity platforms and protective BERs were determined from all 8 PoDs for all exposure scenarios for these chemicals at L2. In the case of chlorpyrifos, the mechanism of action of organophosphates is very well studied and the long-lasting neurological effects are due to inhibition of the acetylcholinesterase enzyme (AChE) as a result of the formation of a chlorpyrifos-oxon (Barron and Woodburn 1995; Richardson 1995). The AChE enzyme is present in the IPP panel, although the current assay set-up does not have inherent metabolic capability to form the oxon metabolite. For chlorpyrifos, a protective BER is still determined from the HTTr data, with differential expression of CYP and UGT genes driving the HTTr PoD in HepG2 cells.

**Table 4.** Summary of the toolbox performance for chemicals with high-risk exposure scenarios and investigation of the ability of the toolbox to make protective decisions irrespective of detection of the mode of action.

Toolbox is not protective	Toolbox is protective but MOA is not detected	Toolbox is protective and MOA detected
Trimellitic Anhydride—serum IgE increase as a result of respiratory sensitization	Azathioprine—purine mimic inhibiting DNA synthesis (potentially metabolism limited)	Cetirizine Hydrochloride—histamine H1 receptor inhibition
Warfarin—VKORC1 inhibition and anticoagulation effects	Benzocaine—methaemoglobin formation	Dexamethasone—Glucocorticoid receptor binding
	Chlorpyrifos—AChE inhibition (metabolism driven)	Fenbuconazole—interference with steroid biosynthesis (Aromatase)
	Cyclophosphamide—DNA binding/damage (metabolism driven)	Furosemide—primary mode of action is Na-k-Cl transporter binding (not detected). Secondary mode of action is increase in prostaglandin synthesis (COX1+COX2) (detected)
	Digoxin—Na^+^/K^+^-ATPase inhibition	Ibuprofen—COX 1 and COX 2 inhibition
	Glibenclamide—K^+^ channel inhibition	Ketoconazole (oral)—prevention of ergosterol synthesis (Aromatase)
	Hydralazine hydrochloride—Ca^2+^ flux interference	Metoclopramide—D2 antagonism
	Metformin—gluconeogenesis inhibition (cellular energy measures respond but at concentrations>exposure)	Paracetamol—COX 1 and COX 2 inhibition (although no metabolic competency in the assay)
	Topiramate—GABA receptor binding (subunit specificity)	Verapamil Hydrochloride—calcium channel blockage

Full details can be found in Table S4.

**Fig. 5.**
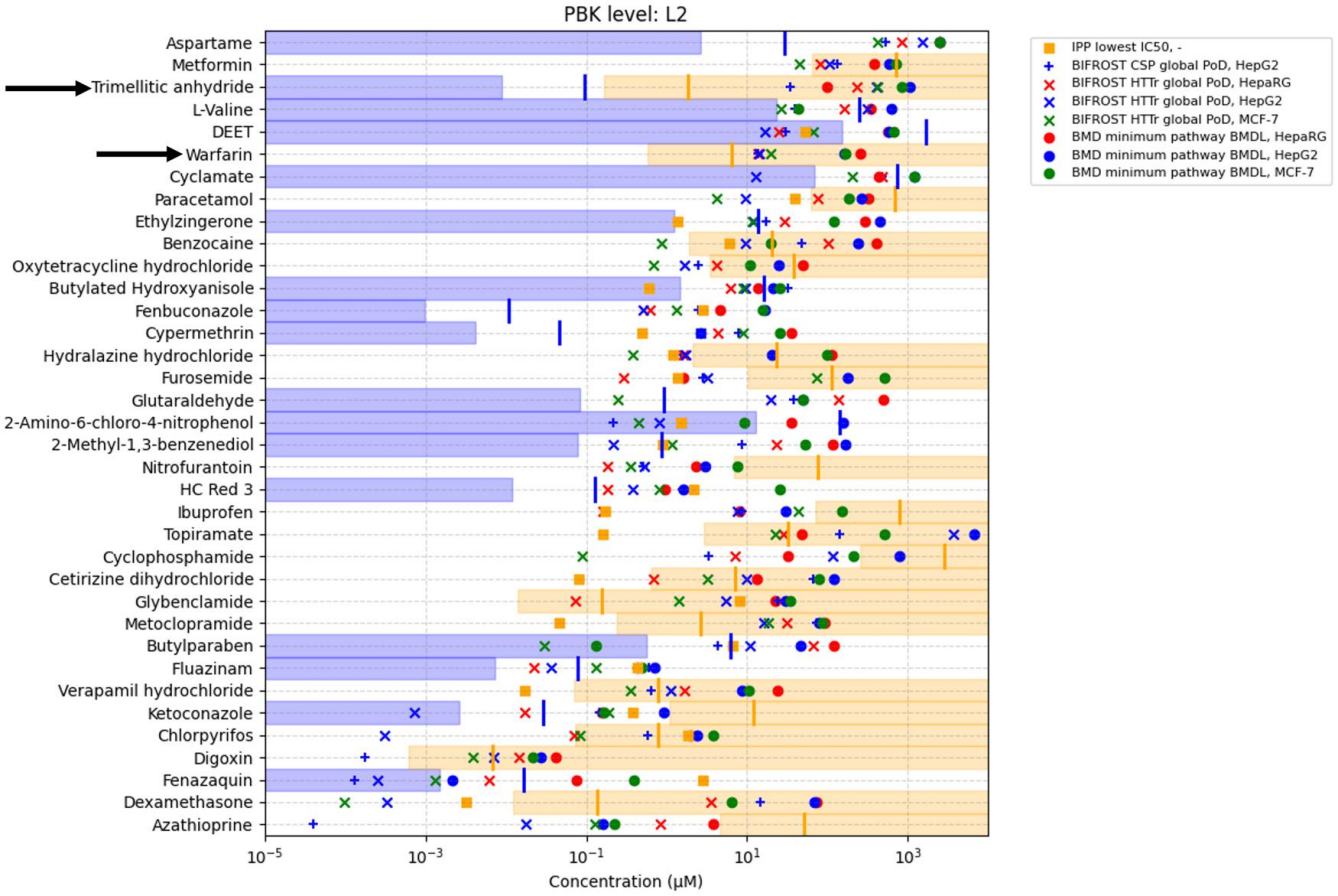
Summary of PoDs for all test chemicals compared with L2 exposure estimates. Blue and yellow horizontal bars represent *C*_max_ estimates for low- and high-risk chemical exposure scenarios, respectively. PoDs calculated from the different bioactivity platforms are shown for each chemical. Vertical lines represent the *C*_max_ multiplied by the L2 BER threshold for each exposure scenario. Where all PoDs are to the right of these vertical lines the toolbox would conclude low-risk, and where there are PoDs that sit to the left of the line (i.e. bioactivity measured at concentrations below exposure) the toolbox would conclude uncertain risk. Black arrows indicate the trimellitic anhydride and warfarin examples where bioactivity is observed at concentrations above the estimated high-risk exposure and therefore a BER>threshold is determined for the scenarios and a protective decision is not made.

Among the chemicals where the mechanism of action was detected (8/38), 3 had a specific mechanism of action that was detected in the IPP panel and there was low bioactivity in the other platforms. Protective decisions for the high-risk exposure scenarios of metoclopramide, cetirizine dihydrochloride, and verapamil hydrochloride rely on the IPP PoDs with other bioactivity platform PoDs calculated to be above the predicted *C*_max_ values. As shown in Fig. 5, the IC50 determined for these chemicals at different targets is the leading PoD and is significantly lower than PoDs determined from the HTTr and CSP. This is particularly evident in the case of metoclopramide, which is a known dopamine antagonist and D2 inhibitor with an IC50 of 0.046 µM in the IPP panel and the next lowest PoD of 16 µM coming from the HepG2 HTTr. Cetirizine dihydrochloride acts on the histamine H1 receptor (Brik et al. 1987) and verapamil hydrochloride acts on calcium channels, although effects are also noted in other receptors with the leading IPP PoD coming from antagonism of the serotonin 2B receptor, which is also reflected in the literature (Auguet et al. 1986; Ali et al. 2016). These examples demonstrate the importance of combining data from different in vitro NAMs measuring both specific and nonspecific effects.

The 15/38 examples of chemicals with low-risk exposure scenarios predicted to be uncertain risk are often scenarios where either only an L1 *C*_max_ prediction is available, or in vitro data for physico-chemical properties only have been used to parameterize the PBK model; meaning that key values such as intrinsic clearance are still predicted instead of being experimentally derived (see Table S2a for details). Increasing the amount of data input to the PBK models should increase the confidence in the accuracy of their predictions. Increasing the accuracy of the PBK models used, plays a significant impact on the performance of the systemic-safety toolbox. There are 5 examples where the decision outcome changes when you move from an L1 to an L2 prediction, all 5 are where the decision changes from uncertain risk to low-risk. As previously stated, L2 predictions are likely to be the most readily available in future scenarios when using NAM-based frameworks to make decisions on new chemicals with no historical information, but there are examples where moving from an L2 to L3 prediction significantly reduces the BER, indicating that the L2 PBK models underpredict the real *C*_max_ values and represent a gap in the current workflow. Exposure scenarios where this occurs include the therapeutic uses of digoxin, warfarin, topiramate, and ketoconazole, with at least a 5-fold underprediction of the L3 result at L2. This is most extreme in the case of digoxin where the L2 *C*_max_ estimate underpredicted the L3 plasma *C*_max_ by ∼30- to 50-fold, the largest of any underprediction, albeit with no change in the safety decision in this case. It is well known that digoxin undergoes active transport within the body and is a substrate of the P-glycoprotein efflux and organic anion transporters. Transporter kinetics are not incorporated into the early tier PBK modelling, but it is possible to do this through the generation of additional in vitro data. Digoxin, warfarin, and topiramate all have a plasma half-life of over 20 h and therefore might require bespoke testing to appropriately parameterize the PBK models.

### Comparison with traditional toxicological data

**Fig. 6.**
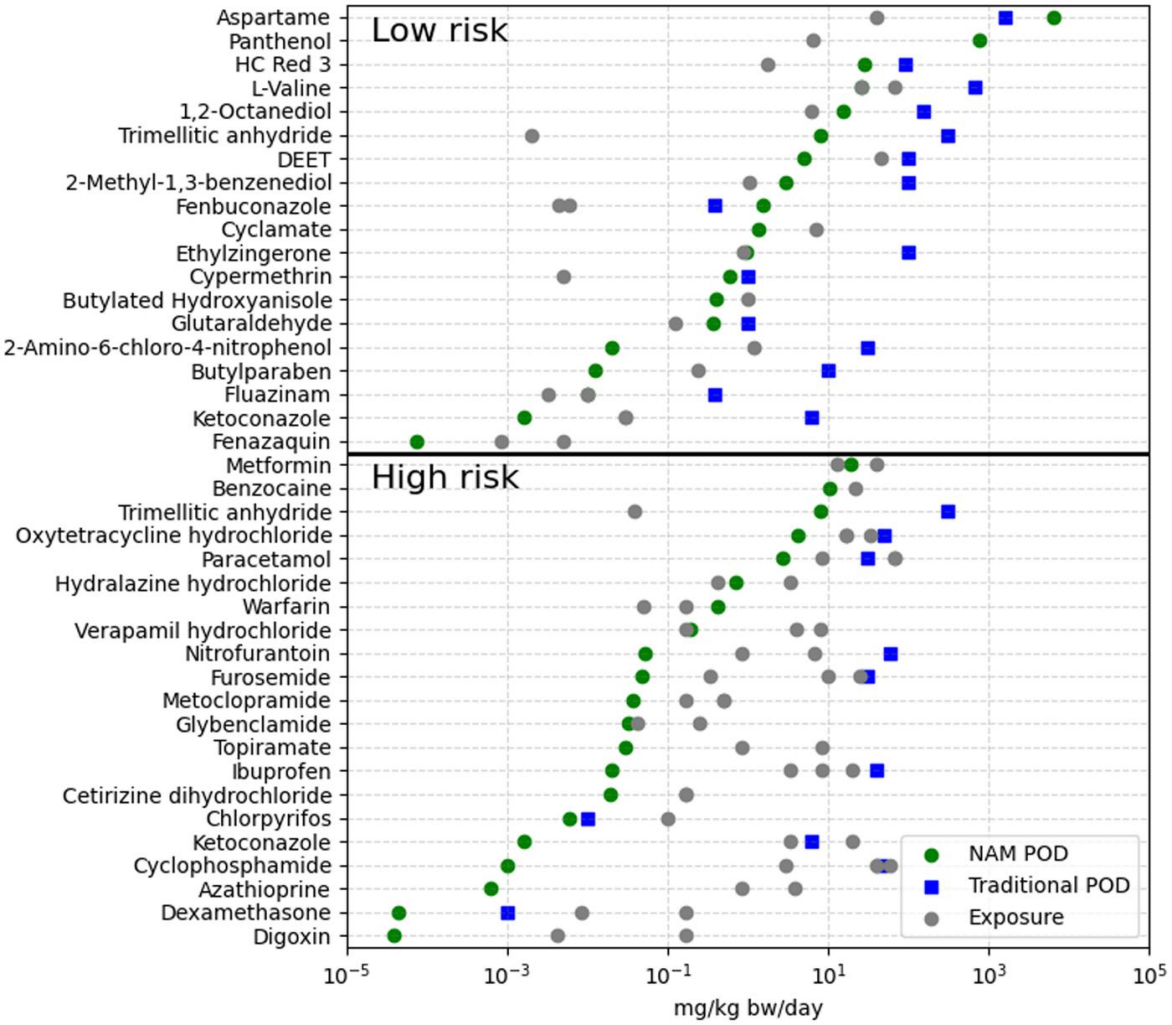
Summary plot of the external exposure estimates with the converted minimum NAM PoDs and traditional PoDs, separated by the risk classification of the corresponding exposure scenarios. Traditional PoDs are only reported for the 25 chemicals where data were available.

A comparison between the NAM-based PoDs and the traditional in vivo PoDs identified from subchronic and chronic animal studies was performed to further investigate the performance of the systemic-safety toolbox. Mammalian in vivo data meeting the criteria set out earlier could only be found for 25 of the test chemicals (see Table S5). In the proposed NAM-based workflow, the use of in vitro data as an early tier uses the minimum PoD identified across all assays performed; so in this comparison, the same approach was taken to the in vivo data where the minimum NOAEL or NOEL identified was taken forward. The Pearson correlation of the traditional and NAM PoDs before and after transformation of the NAM PoD into the equivalent external dose were calculated to be 0.8 and 0.57, respectively (Figs S5 and S6). Like other studies that have compared NAM PoDs with traditional PoDs, we found that for nearly all chemicals the minimum NAM PoD was more conservative, i.e. lower, than the minimum traditional PoD. Exceptions to this were for aspartame and for fenbuconazole, where the difference between the traditional PoD and the NAM PoD was under 5-fold. Figure 6 shows a comparison between these PoDs with the external exposure estimates for the corresponding low- and high-risk exposure scenarios.

**Fig. 7.**
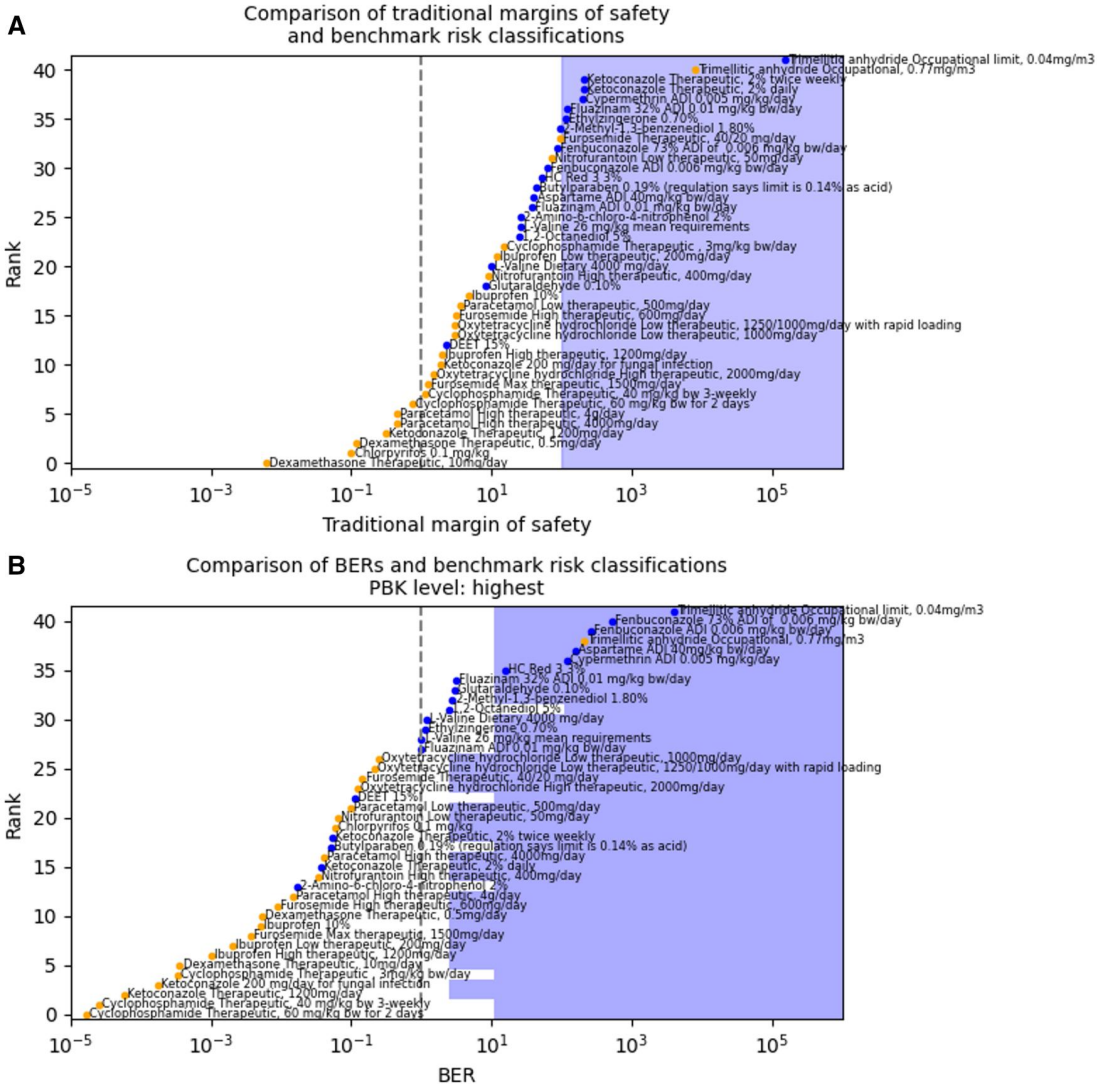
(A) Summary plot of calculated margins of safety for the subset of benchmark chemical exposure scenarios that had traditional animal data available. The blue-shaded region represents a MoS > 100 and therefore likely to be considered low-risk. (B) Summary plot of corresponding BERs for the subset of chemical exposure scenarios. The blue-shaded region indicates a BER>threshold appropriate to the highest parameterization level of PBK model for that scenario.

It should be noted that this direct comparison of the in vitro PoDs and traditional animal PoDs does not factor in the species differences and related assessment factors that are applied when using traditional data in safety decision-making. In order to properly compare the use of traditional data and NAM data for risk assessment, the same performance metrics have been applied to safety decisions made using the traditional in vivo data in the same early-tier approach. External exposure estimates were compared with the minimum PoD from suitable studies and a MoS calculated in the same way that the minimum platform PoD is compared with the plasma *C*_max_ and a BER calculated. To enable the same performance metrics to be calculated an assumed acceptable MoS threshold of 100 was used, as is typically accepted as a suitable MoS (see Fig. 7). The separation of benchmarks is not perfect, even using traditional in vivo data, with overlap of high- and low-risk MoSs. The protectiveness and utility metrics were calculated to be 97% and 42%, respectively, using the threshold of MoS >100. For the same set of chemical exposure scenarios, the systemic safety toolbox had 96% protectiveness and 32% utility. Again, the occupational case study exposure to trimellitic anhydride is misclassified as low-risk using the in vivo data. Unfortunately, no in vivo NOEL or NOAEL could be identified for warfarin, which is to be expected given its potency as a poison in rodents.

## Discussion

Although significant and rapid progress is being made in the development, evaluation, and application of NAMs for NGRA, broad acceptance of these approaches is limited by a traditional validation paradigm. Efforts are being made to update the way in which we build scientific confidence for the use of NAMs in safety assessments, both in regulatory and nonregulatory contexts. Recent publications have shared pragmatic strategies for demonstrating the validity of novel approaches to safety decision-making, identifying key concepts to address to increase the likelihood of regulatory acceptance (Van der Zalm et al. 2022; ICCVAM 2024). Some of these key concepts include a clear definition of the context of use, assessment of the biological relevance, technical characterization, data integrity, information transparency, and an underpinning independent review. We believe this current evaluation establishes many elements of the evidence required to build such scientific confidence.

In this article, we have clearly defined a context of use for this systemic-safety toolbox: Quantitative risk assessment; where the modules comprising the toolbox are used to derive internal exposure estimates, obtain in vitro PoDs, and calculate BERs for a given chemical exposure scenario. This can then be used to either directly support a protective, risk-based safety decision, or identify areas of uncertainty for higher tier investigation. In terms of biological relevance, this is addressed within the toolbox principally by assessing the performance of a multi-NAM approach compared with risk benchmarks anchored in robust traditional toxicology data typically from subchronic 90-d repeat dose studies in test animals, or human data where available. These 70 benchmark chemical exposure scenarios were selected in such a way to maximize the biological and chemical coverage while avoiding any bias that may arise from manual selection, e.g. selecting a set of chemicals that mostly comprises the extremes of bioactivity with no moderately active examples. This bias is potentially reflected in the results of the initial pilot study (Middleton et al. 2022) where perfect separation of the high- and low-risk benchmarks was achieved. Here, the stratified sampling approach allowed for a breadth of chemistry and biological activity to be covered. However, to strengthen the evidence of the toolbox’s ability to make protective decisions based on broad biological activity would need more chemicals to be tested of similar mechanisms of action, or similar effects in humans, or of similar structures, to increase the ability to make conclusions related to specific classes of chemicals. Although we have demonstrated that the test chemicals used in this evaluation do not cover some aspects of the chemical space, other studies have demonstrated the protective nature of in vitro bioactivity lead approaches for chemical prioritization and risk assessment (Chen et al. 2020; Cordova et al. 2023; Tsai et al. 2023; Ford et al. 2024; Tsai et al. 2024). It is also recognized that consistent efforts need to be made as part of carrying out in vitro testing to ensure analytical bias and concordance issues are minimized; separate work has been performed to thoroughly characterize the “true dose” via application of a workflow examining volatility, stability, hydrophobicity, binding to plastic or serum, and solubility of the test material (Nicol et al. 2024).

The systemic-safety toolbox and workflow correctly identified a high proportion (>90%) of high-risk exposure scenarios and appears to offer a similar level of protection as safety assessments based on traditional animal data. Although the default toolbox configuration uses 3 bioactivity platforms that covers specific and nonspecific effects, this is not necessarily the “optimal” configuration from a protectiveness/utility perspective. For this reason, the protectiveness and utility of the toolbox was analyzed under different permutations of the 3 bioactivity platforms. The full toolbox evaluated in this work results in the most conservative overall PoD possible, although the same protectiveness and utility metrics are also observed from combining only the IPP and the HTTr BIFROST Global PoDs. Although the emphasis here has been on the ability to make protective decisions agnostic of a mode of action, mechanistic information can still be assembled from the types of in vitro assays utilized in this work. Furthermore, other studies have demonstrated the ability of in vitro assays to identify additive neurological effects from PFAS/PFOS compounds (Ríos-Bonilla et al. 2024). Although this raises the opportunity presented by in vitro assays to characterize the molecular basis behind observed apical effects and conduct a cumulative risk assessment where appropriate, this remains a developing and challenging area for further work. From the results of this evaluation, in most cases, the identification of a mechanism of action is not required to make a protective safety decision; the exception being warfarin, where the mechanism of action is specific but incorporation of an assay that measures it, could change the risk decision outcome. Other examples where an established and specific mechanism of action were not detected included several examples of chemicals impacting ion flux across membranes, examples where the known mechanism of action relies on the formation of a metabolite and the test systems lack the relevant metabolic competency or examples where specific targets were missing from testing panels. Although these gaps only impacted the protectiveness goal in the case of warfarin, an expansion of the toolbox could look to address some of these effects, or identify a strategy whereby in silico tools in combination with other technologies could be utilized for investigating missing mechanisms of action or alerting the risk assessor to the fact that a relevant mechanism of action might be missing from the toolbox. It must be stressed also that this toolbox is intended to be used as part of a wider risk assessment framework (Fig. 1), and forms only some of the lines of evidence to guide decision-making. To this end, where historical toxicology data are available, they can be used alongside in silico tools to shape the problem formulation stage at Tier 0 and inform whether the use of this toolbox is appropriate. For example, in the case of warfarin, consideration of in silico predictions of acute toxicity would alert the risk assessor that this chemical has a high toxic potency and therefore would be expected to be highly active in vitro. A disparity between the alert for high acute toxicity and the NAM output would lead to a mechanism of action-based evaluation of whether the toolbox sufficiently covered the relevant biology and, if not, additional assays could be considered on a case-by-case basis to represent hypothesized mode(s) of action. This approach, which increases the protectiveness of the toolbox even further, is possible without any existing historical acute toxicity data using robust and reproducible in silico tools that are suitable replacements for animal testing, such as CatMOS (Collaborative Acute Toxicity Modelling Suite) (Mansouri et al. 2021).

As exposure is one of the cornerstones of any toxicological risk assessment, consideration must be given to the performance of the exposure module of the workflow, tested through the modelling of different routes of exposure, different dosing regimens, and different parameterization levels. As expected, the results show that, as you progress through the PBK levels and build models with more human-relevant inputs, the performance of the toolbox improves. However, only a very limited number of low-risk exposure scenarios were associated with chemicals for which clinical data were available, and therefore, the L3 performance is significantly skewed to identifying high-risk scenarios. No conclusions can therefore be drawn on the impact that human kinetic data can have on utility of the toolbox. It is likely that in future, early-tier decision-making will rely on in vitro data for parameterizing the PBK models due to the long timeframes, high costs, and ethical considerations associated with the generation of human clinical data, particularly for novel compounds. Therefore, a relatively high level of confidence is required in PBK models developed using L2 parameterization. Currently, in this evaluation, L2 is defined by the presence of in vitro data for at least one parameter, which can include the experimental derivation of the physico-chemical parameters. It is expected that in future a minimal set of such parameters will be required to include hepatic intrinsic clearance, fraction unbound, and blood:plasma ratio, with additional requirements of parameters such as dermal absorption and intestinal absorption kinetics depending on the route of exposure (Gregoire et al. 2021; Komura et al. 2023; Geci et al. 2024; Wood et al. 2024). A refinement of the toolbox could be to redefine the definition of L2 in the exposure module to include this minimal set of ADME parameters, however, this was not part of the pilot study that set the thresholds for performing this evaluation and would necessitate recalculation of the BER thresholds to address the new definition of L2 before using these to evaluate the toolbox performance with this wider set of chemicals.

After considering the biological relevance of the systemic safety toolbox, moving on to review the technical characterization involves exploring a number of aspects contributing to increasing scientific confidence in the methodologies. One aspect is to develop a suitable statistical data analysis approach. Although this toolbox achieves high protectiveness, a large proportion of low-risk chemical exposure scenarios are identified as uncertain risk, contributing to an overall low utility. At this stage of employing bioactivity-based assays for risk assessment, this low utility is not a surprise as the workflow, as yet, makes no differentiation between bioactivity and *adverse* bioactivity. This is exemplified by the inclusion of markers such as GSH levels and the transcription of metabolism-related genes that may themselves not represent biological effects indicative of toxicity, but further work will be needed to annotate biomarkers specifically relevant for adversity, or thresholds above which observed bioactivity can be called adverse. In this work, the low utility is often driven by the HTTr BIFROST PoDs, where an uncertain risk conclusion would mean progression through to Tier 2 and the use of higher tier models to investigate specific risks on a case-by-case basis. The method for calculating a PoD from transcriptomic data is still under debate, especially when considering the value of using gene-level PoDs or pathway-level ones (Reardon et al. 2023; Harrill et al. 2024), however, in the context of this toolbox, we have shown that using pathway level PoDs as calculated using BMDExpress2 does not enable protective safety decisions to be made in the 3 cell lines tested, and the gene level PoDs calculated using the Bayesian statistical BIFROST model are needed to achieve the maximum protectiveness metrics discussed earlier. In this work, analyzing the data using the BIFROST pipeline required a model to be fit to nearly 1.5 million datasets which is an impractical number to visually inspect the model fit for as recommended in Gabry et al. (2019). Therefore, it is possible that the analysis could falsely infer a gene to be differentially expressed, i.e. result in a false positive in the HTTr data analysis. This could lead to an overly conservative PoD calculation from the transcriptomic data, and as this assay contributes the lowest PoD for 25 of the 38 test chemicals, it could cause overly conservative estimates of bioactivity for a number of the chemicals with low-risk exposure scenarios. An example where this is likely is for panthenol where the HTTr BIFROST HepaRG and MCF7 PoDs are orders of magnitude lower than PoDs determined from other assays and is being led by changes in a single gene in the case of the HepaRG data, and only 2 genes in the MFC7 data (Figs S7–S9). As a result of these transcriptomic data, panthenol is classified as an uncertain risk using the decision model, which is unexpected given the relatively inert nature of panthenol. Future work will address this potential within the statistical modelling for false positives, as even if the false positive rate is low, the large number of datasets analyzed may result in a considerable number of genes wrongly classified as differentially expressed.

Although this article has focussed on the evaluation effort of an integrated NAM-based approach, the proper documentation of NAMs is an important part of ensuring full technical characterization. For example, various activities are underway to reproduce results from the different bioactivity platforms at multiple laboratories to address questions on the reliability of the assays. In addition, principles such as those outlined in the OECD Guidance Document on Good In vitro Method Practices (OECD 2018) have been followed and are being collated to provide details regarding quality practices, equipment procedures, and appropriate documentation are being collated. Sharing details of this approach here, including protocols and code, where it is available for scrutiny by the scientific community, forms a critical part of the information transparency expected for acceptance of new approaches.

Overall, this work adds to the increasing body of evidence showing that NAM-based safety decisions can be protective of human health, and that NAMs can be integrated in a practical and systematic way as part of a tiered approach to enable confident safety decision-making. The approach to evaluating the toolbox also demonstrates that it is possible to assess the performance of a multi-NAM approach by anchoring the evaluation in risk benchmarking.

## Supplementary Material

kfae159_Supplementary_Data
