# Supplementary material for: Advancing systemic toxicity risk assessment: Evaluation of a NAM-based toolbox approach
Source: Toxicol Sci. 2024 Dec 18;204(1):79–95. doi: 10.1093/toxsci/kfae159 (PMC11879040; doi:10.1093/toxsci/kfae159)
Supplement: kfae159_Supplementary_Data [file kfae159_supplementary_data.zip › kfae159_Supplementary_Data/toxsci-24-0360-File010.docx]

## Chemical Selection Process

*Table ST1: Overview of how selection of the test chemicals was performed and the rationale for each step of the process*

| **Step** | | **Details** | **Rationale** |
| --- | --- | --- | --- |
| 1 | Compiling initial list of chemicals. | Input from various databases including the US EPA ToxCast database and test substance lists for the EUToxRisk project [https://eu-toxrisk.eu/], Cosmetics Europe LRSS project [https://www.lrsscosmeticseurope.eu/about-the-lrss/] and expert opinion. | Existing databases were used to encourage selection from a broad set of test chemicals with the aim of maximising the chemical and biological coverage of selected chemicals and aiming to avoid selection bias from relying on expert curation alone. |
| 2 | Filter by practical availability and handling | Automated removal of chemicals where a supplier could not be identified, the cost was prohibitive or the compound was too hazardous to be handled in the relevant labs. | Chemicals had to be able to be sourced and handled as part of the proposed systemic-safety toolbox assays and workflow |
|  | Filter by LogP | Using predicted, or experimentally derived values where available in the literature, all chemicals with a LogP above 7 were removed. The LogP values have been calculated using ADMET Predictor v. 11.0.0.3 [https://www.simulations-plus.com/] | Assay practicalities |
|  | Filter by Molecular Weight | Limit range to substances under 1500 Da and above 80 Da. | Assay practicalities (size suitable for detection by mass spectrometry should work need to be done). Upper limit based on the likelihood of systemic bioavailability. Penetration through the skin or gut is highly unlikely for compounds with a molecular weight over 1500 Da [Bos et al., 2000]. |
| 3 | Stratify by use categories | Using CPCat use annotations, all ~1700 compounds were put into 5 ‘bins’. Annotations were manually curated into 5 distinct use-case categories: Cosmetic, Food, Drug, HomeCare and Agriculture. There were some remaining that were left as Miscellaneous although these were mostly industrial/automotive categories that would be less likely to result in human exposure and therefore no chemicals were sampled from the miscellaneous category. | In order to aid unbiased randomised selection whilst ensuring a spread of biological activity. |
| 4 | Shortlist generated from bins | 40 from each category were selected giving a total shortlist of 196 compounds.  N.B. Only 36 compounds were identified with a cosmetic use annotation whilst 40 were identified in other categories. | As a key process was to identify an exposure scenario, a shortlist was created to account for the likelihood that an exposure scenario would not be found for some chemicals and replacements would be needed. |
|  | Exposure Scenario identification | Search terms and website/database links were provided to a third party to conduct a search to identify chemicals were at least 1 consumer exposure scenario could be defined.   - Drugbank [https://go.drugbank.com/] - Joint FAO/WHO Expert Committee on Food Additives (JECFA) [https://apps.who.int/food-additives-contaminants-jecfa-database/] - Cosmetic Ingredient Review (CIR) [https://www.cir-safety.org/] - European Food Safety Authority (EFSA) [https://www.efsa.europa.eu/en/publications] - EU Pesticides [https://food.ec.europa.eu/plants/pesticides/eu-pesticides-database_en] - EPA Comptox Chemicals Dashboard [https://comptox.epa.gov/dashboard/] - EU Cosmetics regulation annexes II and III and IV [https://ec.europa.eu/growth/tools-databases/cosing/reference/annexes] - The Scientific Committee on Consumer Safety (SCCS) and Scientific Committee on Cosmetic Products and Non-food products (SCCP) opinions [https://health.ec.europa.eu/scientific-committees/former-scientific-committees/scientific-committee-consumer-safety-2016-2021/sccs-opinions-2016-2021_en] - Examine [https://examine.com/] - UK Clinical Trials database [https://clinicaltrials.gov/] - Joint FAO/WHO Meeting on Pesticide Residues (JMPR) [https://www.who.int/groups/joint-fao-who-meeting-on-pesticide-residues-(jmpr)/publications/reports] - Drinking water guidelines [https://www.who.int/teams/environment-climate-change-and-health/water-sanitation-and-health/water-safety-and-quality/drinking-water-quality-guidelines] - European Medicals Agency (EMA) [https://www.ema.europa.eu/en/homepage] - U.S. Food and Drug Administration (FDA) [https://www.fda.gov/drugs/development-approval-process-drugs/drug-approvals-and-databases] - Dutch National Institute for Public Health and the Environment (RIVM)[ https://www.rivm.nl/en] - Australian Industrial Chemicals Introduction Scheme (AICIS, formerly: NICNAS)[ https://www.industrialchemicals.gov.au/] - European Chemicals Agency (ECHA)[ https://echa.europa.eu/information-on-chemicals] | Systematic process developed to search for exposure scenario information. Chemicals with no identified exposure or with an exposure scenario that was hard/impossible to quantify were removed.  E.g. chemicals with exposure scenarios that would be difficult to model using PBK were removed such as Chloramphenicol administered via ocular route. |
|  | Assignment of risk category to exposure scenario | The high-risk/low-risk decision for each chemical exposure scenario was made from the perspective of a consumer good. Full details of the risk classifications and rationales for decisions can be found below. | To be used as benchmark chemical exposure scenarios in the evaluation of the performance of the toolbox assays, a high-risk/low-risk decision had to be made using existing data for each scenario. |
| 5 | Chemical Space profiling | A projection of chemical space was created based on the physico-chemical properties of each shortlisted chemical. Based on a principal component analysis (PCA) visualisation of these, the selection of 38 of the most structurally diverse chemicals covering each use and risk categories was undertaken. | One of the methods used to ensure maximum structural diversity in the range of chemicals used to evaluate the toolbox. The breadth of chemical space covered by the shortlisted chemicals that met the minimum data requirements was assessed and used to guide decisions on the final selection. |
|  | Biological activity profiling | Literature searches were conducted for the chemicals to identify likely modes of action/mechanisms of toxicity and to record the different target organs or adverse effects resulting from chemical exposure. ADI/TDI or NO(A)ELs/LO(A)ELs from key studies were also collated. There were some gaps for certain drugs as NO(A)EL/LO(A)EL values were not identified from literature. | One of the methods used to ensure diversity in the range of chemicals tested. The final selection was intended to maximise the different adverse effects/targets organs recorded in the literature for the different chemicals. Information on reference doses or *in vivo* points of departure were used as an indication of the breadth of chemical potency covered. |
| 6 | Selection of Final Test Chemicals | Multiple expert curation of the shortlist to span the different known biological effects (and choice made in concordance with the chemical space mapping to ensure representation across the chemical and biological space. | Total test number was guided by experimental practicalities in plate design/assay set up. |

## Computing Chemical Space – first reduction stage details

Several metrics can measure the diversity of a set of objects, and Shannon’s Entropy is one of the most common (Jost, L. Entropy and diversity. *Oikos* **113**, 363–375 (2006)). A specific method, already reported in the literature (Contreas et al., ACSAppl. Mater. Interfaces 2023, 15,14155−14163, https://doi.org/10.1021/acsami.2c23182) was applied to measure diversity of descriptor columns; such method consisted in measuring the ratio between Shannon’s Entropy of the descriptor vector and the Shannon’s Entropy of an ideal descriptor vector. For example, let $d_{real}$ and $d_{ideal}$ be two descriptor vectors, where the former is the actual descriptor vector, and the latter is the descriptor vector whose values all have the same frequency:

$$d_{real}= \left[ 1,1,0,0,0,0,0,0,0,0 \right]$$

$$d_{ideal}= \left[ 1,1,1,1,1,0,0,0,0,0 \right]$$

The Shannon’s entropy of a vector $x$ can be measured as follows:

$$S_{x}= -\sum_{i=1}^{n} P\left( x_{i} \right)\ln P\left( x_{i} \right)$$

For $d_{real}$, that has 20% of its values being 1 and 80% being 0, its entropy will be:

$$-\left( 0.2\ln\left( 0.2 \right) \right)-\left( 0.8\ln\left( 0.8 \right) \right)=0.321+0.178=0.499$$

However, its ideal entropy would be:

$$-\left( 0.5\ln\left( 0.5 \right) \right)-\left( 0.5\ln\left( 0.5 \right) \right)=2*0.347=0.694$$

As all values in $d_{ideal}$ have the same frequency. The diversity value of $d_{real}$ is given by the ratio between these two entropies:

$diversity = \frac{S_{real}}{S_{ideal}}=\frac{0.499}{0.694}=0.719$

So, filtering descriptors by their diversity ensures that they have values that occur with an acceptable frequency across the dataset. This is important especially for pseudo-categorical descriptors having a limited number of possible values, such as number of rotatable bonds, oxygens and so on.

## Conversion of NAM PoDs from µM to mg/kg bw/day

NAM PODs are converted from their natural units of µM to external mg/kg bw/day equivalents. This is done for each modelled exposure scenario under the assumption that there is a linear relationship between the external dose (mg/kg bw/day) and a measure of the internal of the internal Cmax (µM). Under the assumption of a linear relationship between external dose and internal Cmax, given a single Cmax estimate $C\left[ \mu M \right]$ computed at external dose $E\left[ \mathrm{mg}/kg bw/\mathrm{day} \right]$, the general relationship between external dose $x \left[ \mathrm{mg}/\mathrm{kgbw}/\mathrm{day} \right]$ and internal dose $y \left[ \mu M \right]$ is described by the equation

$$y =\alpha x \text{where} \alpha[\mu M kg bw day/mg]=\frac{C \left[ \mu M \right]}{E \left[ \mathrm{mg}/kg bw/\mathrm{day} \right]}.$$

The inverse of this equation may be used to map NAM PODs, expressed in units of molar concentration, to an external equivalent. Let $P \left[ \mu M \right]$ be the NAM POD for a chemical whose exposure scenario is under consideration. Then, the external equivalent POD, $Q \left[ \mathrm{mg}/kg bw/\mathrm{day} \right]$ is calculated as

$$Q \left[ \mathrm{mg}/kg bw/\mathrm{day} \right]=\frac{P \left[ \mu M \right]}{\alpha[\mu M kg bw day/mg]}=\frac{P \left[ \mu M \right]E \left[ \mathrm{mg}/kg bw/\mathrm{day} \right]}{C \left[ \mu M \right]}.$$

A schematic of this procedure is presented in Figure 1. It should be noted that the mapping of internal PODs to external PODs is dependent on the exposure scenario under consideration. For chemicals with more than one exposure scenario, this results in a one-to-many relationship after performing the conversion. This dependency on exposure route is intuitive, since, for an arbitrary chemical, it would be unreasonable to expect that a dermal exposure at $X$ mg/kg bw/day is inherently the same with respect to internal bioactivity and risk as, say, an oral exposure at the same $X$ mg/kg bw/day.


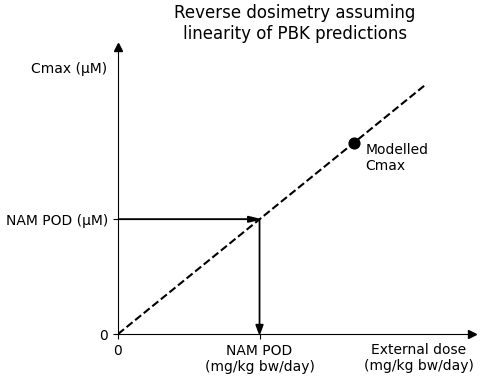


Figure S1 Schematic of the reverse dosimetry procedure assuming linear relationships between PBK Cmax estimates and external doses

## BER Plots with L1, L3 and Highest available PBK predictions


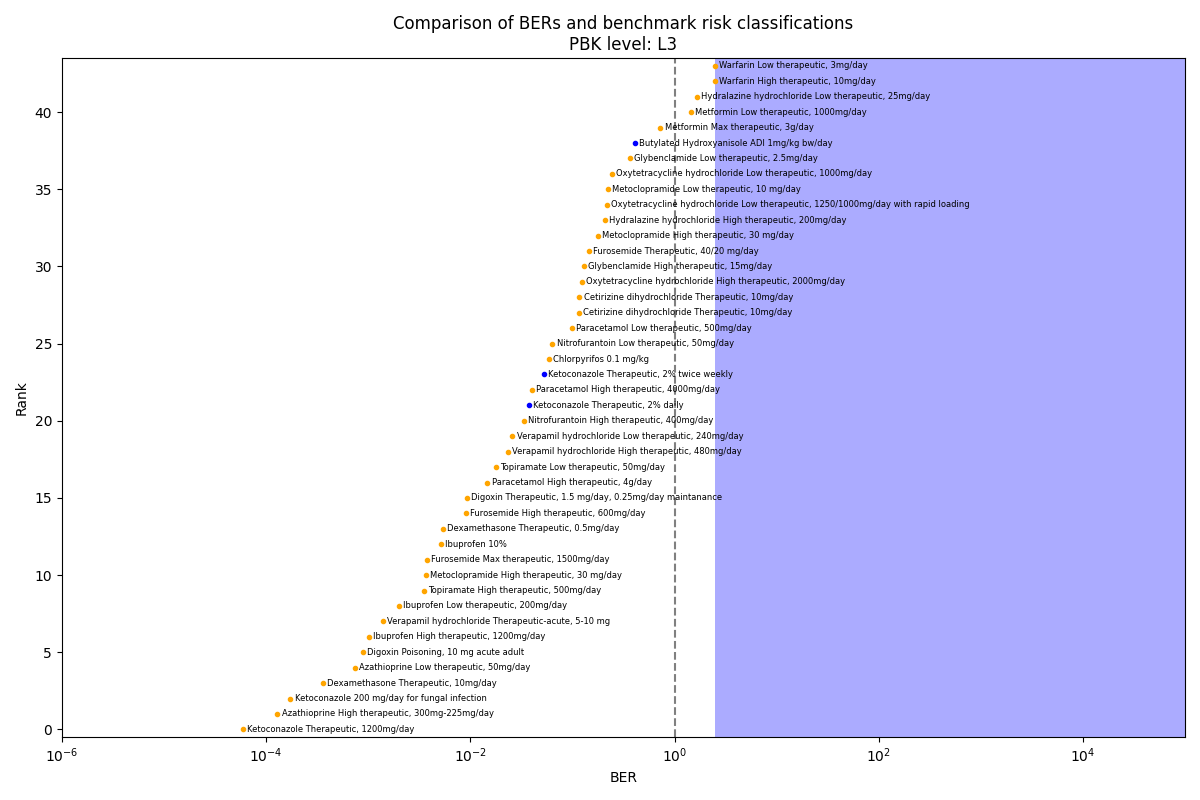

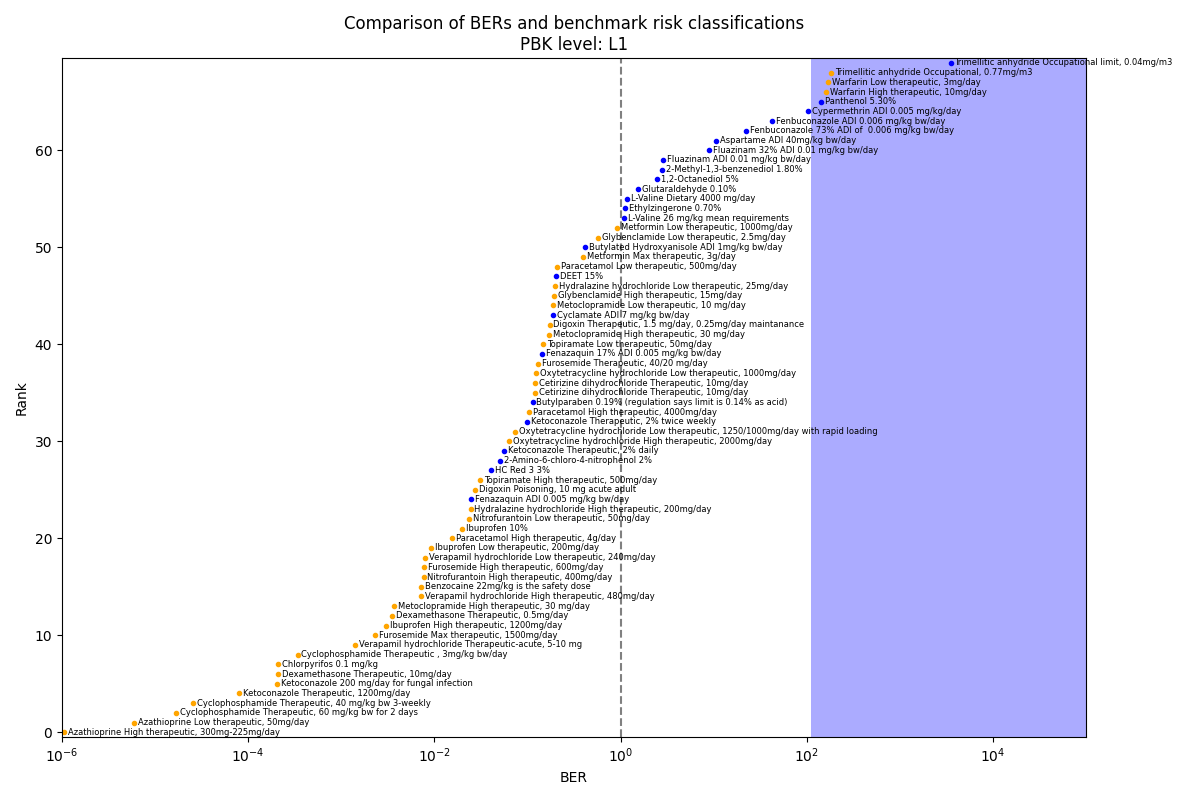


Figure S3 BERs for all evaluation chemicals with an L3 Cmax estimate

Figure 2 BERs for all evaluation chemicals using L1 Cmax estimates.


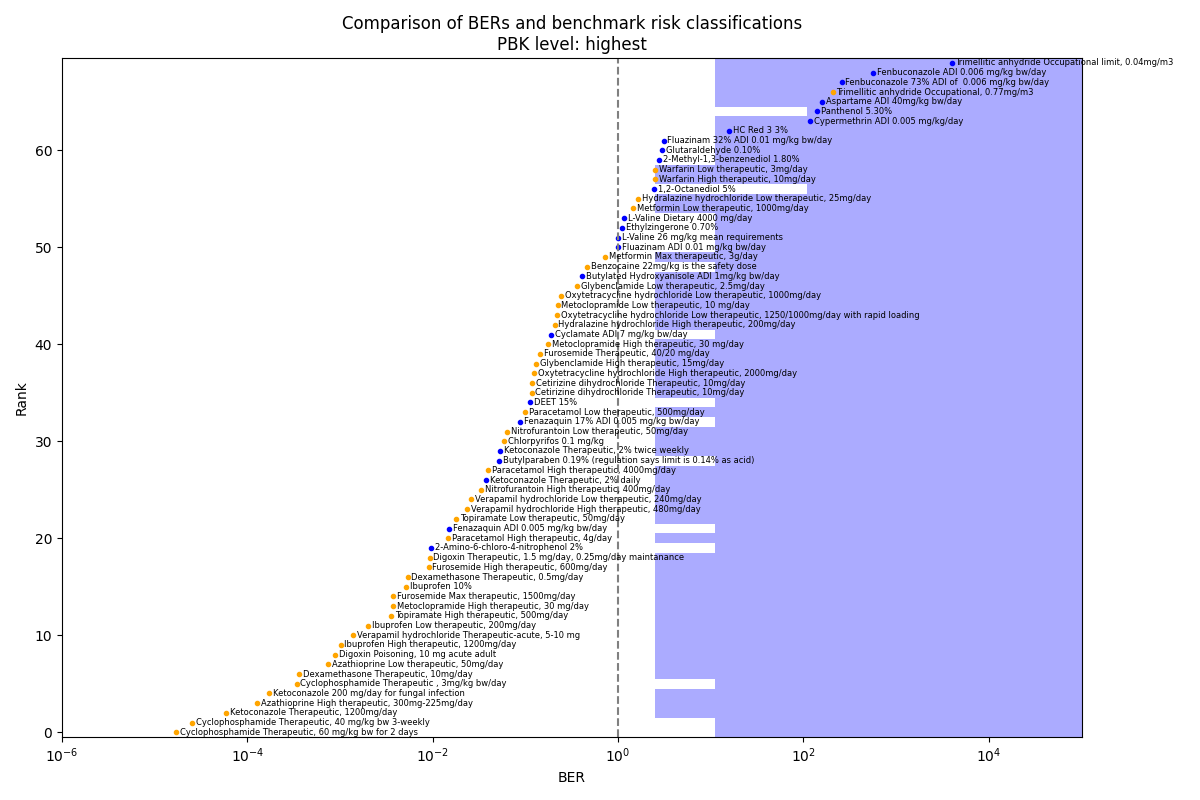


Figure S4 BERs for all evaluation chemicals using the highest available Cmax prediction. The shaded region represents the respective BER threshold for the PBK level at each chemical exposure scenario.

## Correlation of traditional and NAM PoDs


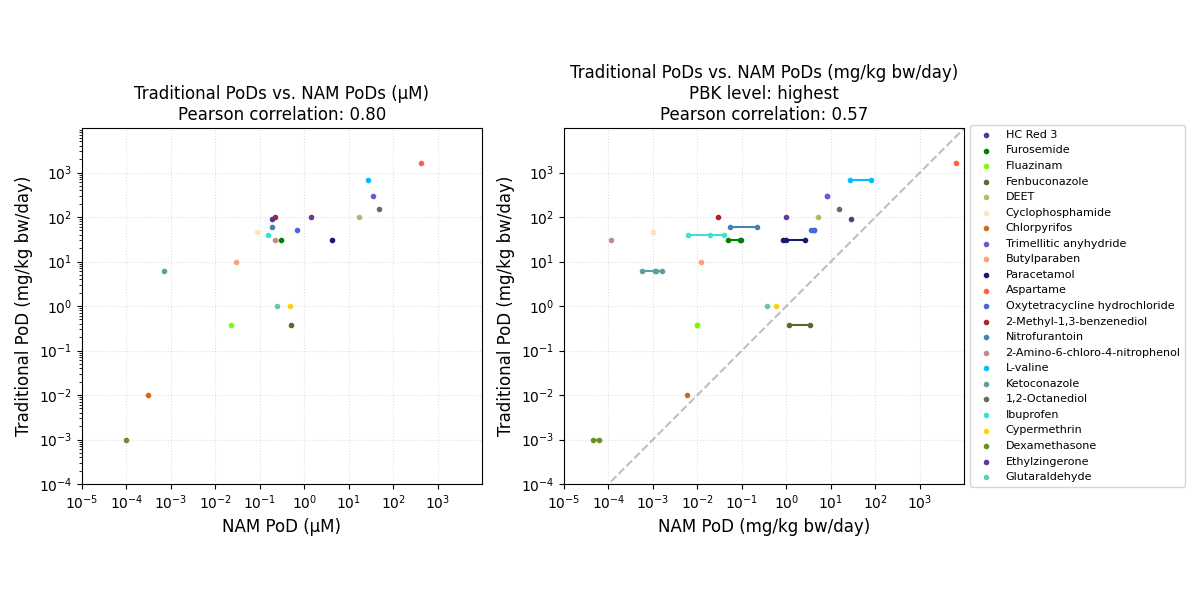


Figure S5 Correlation of traditional PoDs in mg/kg bw/day with untransformed NAM PoDs (uM). Colour coding in legend for Fig. 6


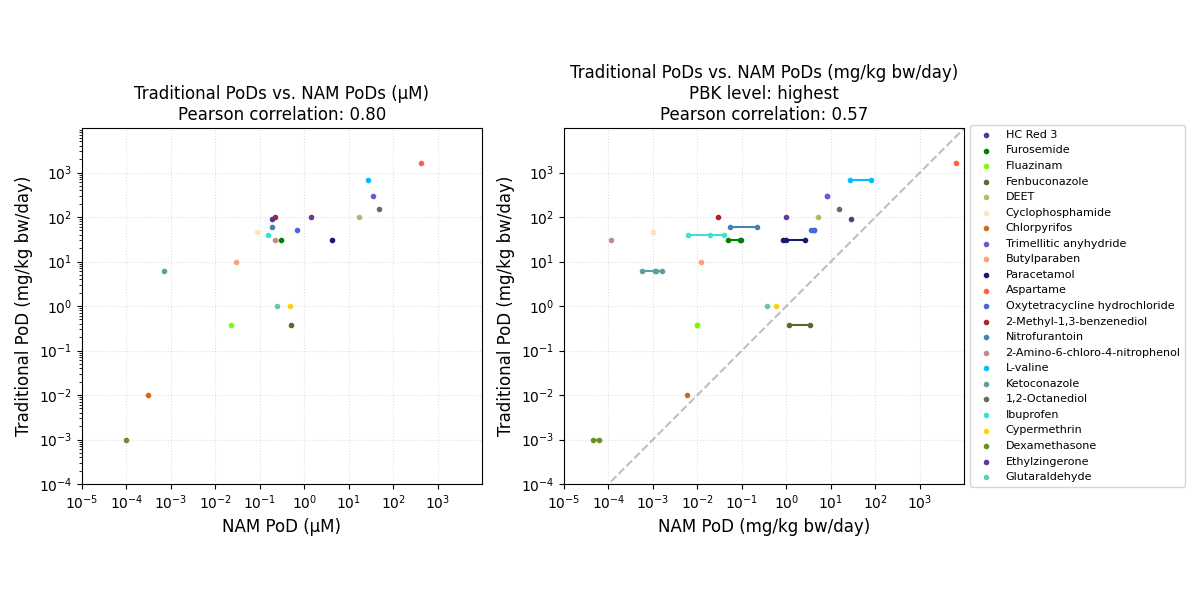


Figure S6 Correlation of traditional PoDs with transformed NAM PoDs. Grey dashed line represents where the traditional PoD=NAM PoD. All points to the left of the line are traditional PoD > NAM PoD. Multiple points are plotted for some chemicals where there are multiple PBK models built and therefore the NAM PoD has been transformed using each of them.

HTTr results for Panthenol


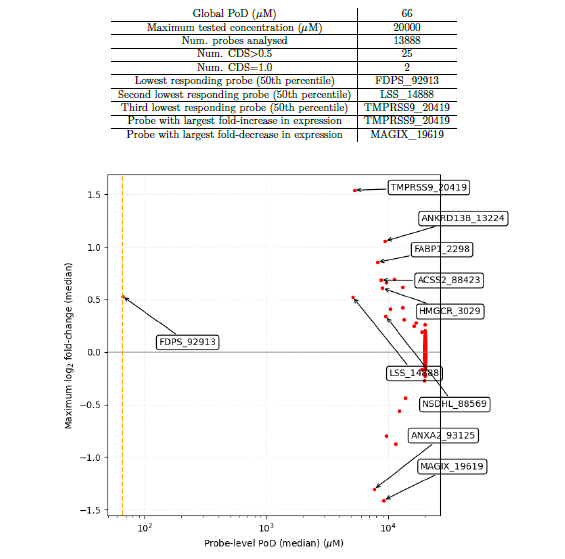


Figure S7 Maximum fold-change in expression in HepaRG cells over the tested concentration-range plotted against the probe-level PoD (median). The orange vertical dashed line is plotted at the nominal global PoD.


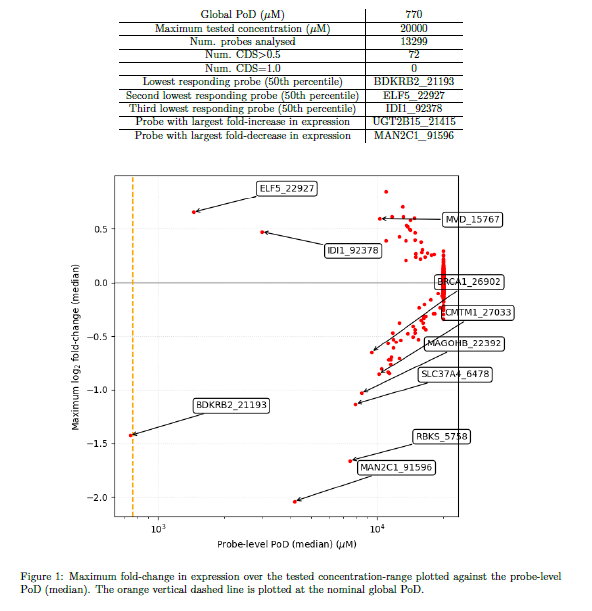


Figure S8 Maximum fold-change in expression in MCF-7 cells over the tested concentration-range plotted against the probe-level PoD (median). The orange vertical dashed line is plotted at the nominal global PoD.


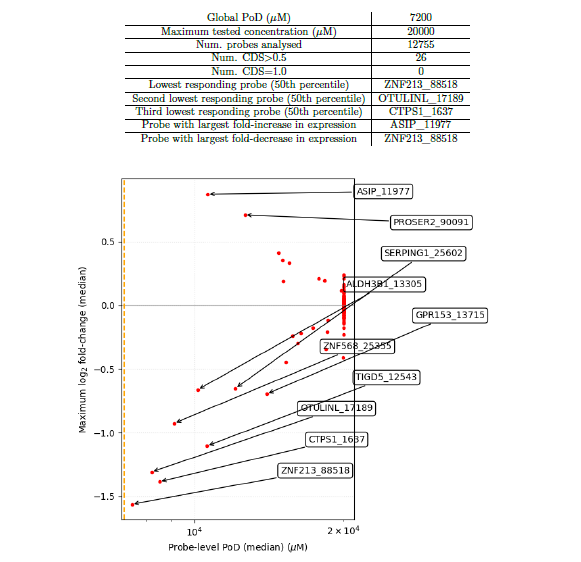


Figure 9 Maximum fold-change in expression in MCF-7 cells over the tested concentration-range plotted against the probe-level PoD (median). The orange vertical dashed line is plotted at the nominal global PoD.
